# A Reference Genome of *Bursaphelenchus mucronatus* Provides New Resources for Revealing Its Displacement by Pinewood Nematode

**DOI:** 10.3390/genes11050570

**Published:** 2020-05-19

**Authors:** Shuangyang Wu, Shenghan Gao, Sen Wang, Jie Meng, Jacob Wickham, Sainan Luo, Xinyu Tan, Haiying Yu, Yujia Xiang, Songnian Hu, Lilin Zhao, Jianghua Sun

**Affiliations:** 1 State Key Laboratory of Integrated Management of Pest Insects and Rodents, Institute of Zoology, Chinese Academy of Sciences, Beijing 100101, China; wushuangyang@ioz.ac.cn (S.W.); mengjie@ioz.ac.cn (J.M.); jacobwickham@ioz.ac.cn (J.W.); xiangyujia@ioz.ac.cn (Y.X.); zhaoll@ioz.ac.cn (L.Z.); 2 State Key Laboratory of Microbial Resources, Institute of Microbiology, Chinese Academy of Sciences, Beijing 100101, China; shenghan.gao@foxmail.com (S.G.); wangsenh86@caas.cn (S.W.); luosn@im.ac.cn (S.L.); tanxy@im.ac.cn (X.T.); yuhy@big.ac.cn (H.Y.); husn@im.ac.cn (S.H.); 3 University of Chinese Academy of Sciences, Beijing 100049, China; 4 CAS Center for Excellence in Biotic Interactions, University of Chinese Academy of Sciences, Beijing 100049, China

**Keywords:** *Bursaphelenchus mucronatus*, *Bursaphelenchus xylophilus*, Comparative genomics, GPCR

## Abstract

The *Bursaphelenchus mucronatus*, which was highly similar with *Bursaphelenchus xylophilus* in terms of morphological characteristics and biological properties—but had weaker pathogenicity to forests—was a native species often displaced by *B. xylophilus* when occupying the same niche. Since the draft genome of the invasive *B. xylophilus* has been published, the absence of a reference genome of *B. mucronatus* still prevents us from understanding the molecular evidences behind competitive displacement. In this study, we employed Single Molecule, Real-Time (SMRT) sequencing and a Hi-C scaffolding approach to yield a near chromosome-level assembly of *B. mucronatus*, including six pseudo-chromosomes. The assembly size is 73 Mb, with scaffold N50 of 11.50 Mb and contig N50 of 1.48 Mb. Comparative genomics results showed high similarity between *B. xylophilus* and *B. mucronatus*. However, the losing of orphan genes and species-specific orthologous genes in *B. mucronatus* may indicate weaker adaptability to the environment. The gene family contractions of GPCRs (G Protein-Coupled Receptors) and cellulases in *B. mucronatus* may jointly contribute to its displacement by *B. xylophilus*. Overall, we introduced a valuable genomic resource for molecular and evolutionary studies of *B. mucronatus*, especially for studying the competitive displacement by the pinewood nematode, which could help us control the pathogenicity of pine wilt diseases.

## 1. Introduction

The pinewood nematode, *Bursaphelenchus xylophilus*, was introduced from North America to Asia and Europe; it is now a global quarantine pest and the main cause of pine wilt disease. It has caused severe disaster to coniferous forest ecosystems [1,2]. In contrast with *B. xylophilus*, there was another native sympatric sibling species, *Bursaphelenchus mucronatus*, which was highly similar with *B. xylophilus* in terms of morphological characteristics and biological properties [3], but has weaker pathogenicity to forests [4,5,6,7]. A previous study showed that the juveniles and males of the two nematode species are difficult to distinguish morphologically, although the tail of adult female worms between *B. xylophilus* and *B. mucronatus* are different. Specifically, the tail of female *B. mucronatus* is conical and has a distinct tail tip [6,8], whereas *B*. *xylophilus* has a rounded terminus [8]. They also occupy the same niche, reproduce in the resin canals, feed on epithelial cells in living hosts, consume the host’s symbiotic fungi in dead hosts, and are even transported to host pine trees by the same insect vectors, *Monochamus* beetles [4,9]. Interestingly, even the two nematodes are highly similar and reside usually in the shared ecosystems; some evidences indicate that *B. xylophilus* usually displace the native nematode *B. mucronatus* in forests during its invasion process [10,11]. Therefore, previous work, which mostly focused on comparisons of biological characteristics (morphology, pathogenicity, reproduction, etc.) between *B. xylophilus* and *B. mucronatus* [7,11,12,13] could not sufficiently explain why and how the competitive replacement took place.

Genomic resources have proven to be useful tools to explain the successful expansion of invasive species. The *Cydia pomonella* genome reveals the genetic basis of its invasiveness with three single nucleotide polymorphisms (SNPs) found in the promoter of CYP6B2. These are potentially associated with insecticide resistance that may explain its worldwide expansion [14]. The *Hyphantria cunea* genome infers that the fall webworm can colonize novel hosts, mediated by plasticity in their gustatory receptor, which could utilize more novel nutrition sources [15]. The genome of the invasive pest *Lycorma delicatula* has served as a reference for studying olfactory genes to restrict its expansion on the fruit or horticultural crops [16]. The comparative genomics study of four *Strongyloides* species exploited the unique *Strongyloides* life cycle and found the expansion gene family of astacin-like and SCP/TAPS (Sperm-coating protein (SCP)-like extracellular proteins, also called SCP/Tpx-1/Ag5/PR-1/Sc7) is associated with the evolution of parasitism [17]. Given these golden examples, the comparative genomic analysis between *B. xylophilus* and *B. mucronatus* is expected to be a key to the answers. Since the draft genome of the invasive species *B. xylophilus* has already been published [18], the absence of a reference genome of *B. mucronatus* still prevents us from understanding the molecular evidences behind this competitive displacement, or even, much simply, are the two nematodes the same species or not? A reference genome for *B. mucronatus* is a necessary resource and a better way to understand the competition between the two species.

Thus, we hereby have sequenced and assembled a reference genome for *B. mucronatus* using PacBio SMRT (Single Molecule, Real-Time) technology, and then carried out a comparative genomic analysis with the previously released *B. xylophilus* reference genome. This reference genome is to investigate evidences for the evolution, the pathogenicity, and the competitive displacement between *B. xylophilus* and *B. mucronatus*, which may help us better understand the pathogenesis of pinewood nematode and control the pine wilt disease.

## 2. Materials and Methods

### 2.1. Sample Collection, Cultivation, and Growth Synchronization

The nematode samples of *B. mucronatus*, which originated from Zhejiang Province, China, were cultivated on the fungus *Botrytis cinerea* using potato dextrose agar (PDA) plates. About 100 adult nematodes were added to the PDA plate with *B. cinerea*. Moreover, 1X PBST buffer (Phosphate Buffer Saline Tween-20) was used to wash out the nematodes after feeding on *B. cinerea;* the suspension was poured into a clean glass petri dish and stored for 30 min. The excess suspension was poured out when most of the eggs were adsorbed to the bottom of the glass. Sterile distilled water was added to the glass to form a water layer with a height of about 5 mm, and cultured in the dark at 25 °C until most of the eggs hatched and became the second instar. Then, the second instar larvae (L2) were cultured with *B. cinerea*. After 24 h–30 h, L2 grew into the third instar larvae (L3); after 48 h–54 h, L2 grew into the fourth instar larvae (L4); and after 72 h–78 h, L2 grew into the adults. Different stages of nematodes were collected by the Baermann funnel method.

### 2.2. Library Construction and Sequencing

Total genomic DNA was extracted from about 20,000 adult nematodes of mixed age using MagAttract HMW DNA Kit (QIAGEN, Valencia, CA, USA), according to the manufacturer’s protocol. Agarose gel electrophoresis was used to check the average length of genomic DNA molecules; the main band was around 20 kb, which satisfied the requirement of assembling sequences with PacBio long read. SMRTbell™ Template Prep Kit was subsequently used to construct the library for the PacBio RS II platform (Pacific Biosciences of California, Menlo Park, CA, USA). A total of 24 single-molecule real-time cells were produced with P6/C4 chemistry, generating 720 million reads with a total length of 11 Gb subreads, with a coverage of 160x of the *B. mucronatus* genome. Meanwhile, a paired-end library with an insert size of 300 bp was constructed using KAPA Hyper Prep Kit (KK8504) and then sequenced on HiSeq 4000 platform (Illumina, San Diego, CA, USA), which was used for assembly survey and assembly correction.

To anchor the assembled sequences into chromosomal level pseudo-molecules, a Hi-C library was constructed using another batch of 20,000 nematodes of mixed stage. Nuclei were extracted and subsequently digested with HindIII and DpnII enzymes, incubated with biotin-14-dATP, and then ligated by T4 DNA polymerase. The extracted DNA was sheared by Proteinase K and purified to 350 bp sizes. Magnetic beads were used to conduct biotin pull down and tail the adapter. PCR amplification was performed after adapters were ligated to the Hi-C products. The PCR products were purified with AMPure XP beads, and the Hi-C libraries were quantified by qPCR for Illumina HiSeq X-ten PE150 sequencing.

Five transcriptomic libraries of *B. mucronatus* in different life stage (Adult, L2, L3, and L4, mixed age) was built by KAPA Stranded mRNA-Seq Library Preparation kit (KK8421) and quantified by qPCR for Illumina HiSeq 4000 PE150 sequencing. The mixed age of transcriptomic library of *B. xylophilus* was built using the same method and the same cultivation method as *B. mucronatus*. Note that these age-specific RNA sequencing (RNA-seq) libraries were designated for constructing transcripts for training and predicting coding genes; thus, no biological replicates were employed.

### 2.3. Genome Size Estimation

The genome size was estimated based on the K-mer frequency distribution counted from Illumina pair-end libraries. The K-mer frequency was counted by Jellyfish [19] with parameter “-m 17 -C” and the result was uploaded to GenomeScope [20] web portal to estimate the genome size. For the GenomeScope parameter, we used a 4-peak model that converged in 4 rounds. The estimated genome size was around 67.5 Mb (Table S1).

### 2.4. Genome Assembly

A total of 11 Gb (~160X) whole genome shotgun PacBio P6/C4 reads generated from PacBio RS II platform were used for genome assembly (Table S2). We used Canu (version 1.8) [21] to generate the de novo assembly of *B. mucronatus*. To obtain an optimal result, we separated the assembling progress into two stages. First, we generated the corrected long reads using Canu with default parameters. Second, we carried out a series of runs using the corrected reads with different combinations of parameters, in the hope of searching for better integrity (higher N50 length and fewer numbers of contigs) of the genome assembly (Table S3). Finally, we chose the parameter combination of “minReadLength=2000, minOverlapLength=2000, correctedErrorRate=0.06, batOptions=-dg 3 -db 3 -dr 1 -ca 500 -cp 50” for the assembly. The assembled contigs were further corrected by Illumina pair-end reads using Pilon [22] with default parameters. The initial assembly contained 1,030 contigs, which sum up to 83,092,420 bp (83 Mb), including 28 repeats with a total length of 606,152 bp. The N50 length of the assembly was 1.48 Mb and the L50 number was 12 (Table S4).

### 2.5. Pseudo-Chromosomal Level Scaffolding with Hi-C Data

The reads from the Hi-C library (Table S5) sequencing were mapped to the assembled contigs using Juicer [23], visualized using Juicebox [23] and anchored into pseudo-chromosome using 3D-DNA pipeline [24]. Juicebox Assembly Tools (JBAT) [23] was used to manually polish and finalize the assembly. Finally, we obtained six pseudo-chromosomes and assigned the chromosome number by their sorted length. In addition to the pseudo-chromosomes, there remains 66 unplaced contigs (Sequence length>=30 Kb) (Table S6).

### 2.6. Genome Annotation

#### 2.6.1. Protein-Coding Gene Annotation

Protein-coding genes were annotated by integrating transcriptomic evidence (assembled transcripts from RNA-seq) with the result from ab initio gene predictors and homologous mapping. Four stages of RNA-seq data were respectively mapped to the genome using Hisat2, and transcripts were constructed using stringtie [25]. The transcripts from all stages were further combined using TACO [26]. Transdecoder was then used to extract candidate ORFs (Open Reading Frame) from TACO results [27]. PASA (version 2.3.3) [28] was used to generate a training set for ab initio gene predictors. Augustus (version 3.3) [29] and GeneMark-ET (version 2.3) [30] were used for ab initio gene prediction. All predicted ORFs were integrated with EVM (EVidenceModeler) [31] and polished with GFFread [32] as the final set of protein-coding genes. All of these protein-coding genes were characterized for their putative function against public database by BLASTP (BLAST: Basic Local Alignment Search Tool for Proteins) (1e-5), including NR (Non-Redundant Protein Sequence Database) [33], UniProt [34], and Kyoto Encyclopedia of Genes and Genomes (KEGG) databases [35]. Meanwhile, InterProScan was used to align protein-coding genes against the InterPro database [36] to identify protein domains. Completeness of protein-coding genes was evaluated by the BUSCO pipeline (Version 3.0.2, lineage dataset is: nematoda_odb9) [37] using both the proteins and genome mode, which may also asses the quality of the assembly. The final assembly of *Bursaphelenchus mucronatus* has been deposited in GenBank with accession number of VUOU00000000. Raw reads are deposited in the Sequence Read Archive (SRA) database under BioProject accession PRJNA562094, including RNA-seq, genome survey sequencing and PacBio sequencing.

#### 2.6.2. Repeat and Non-Coding RNA Identification

RepeatModeler (version 1.0.5, http://www.repeatmasker.org/) was used to construct a species-specific repetitive sequence database. RepeatMasker (version 4.0.5, http://www.repeatmasker.org/) was subsequently used (WUBLAST engine, default parameters) to identify repetitive sequence using database from RepeatModeler.

The rRNA, miRNA, and spliceosomal RNA genes were identified using rfam_scan (version 1.2) with known sequence from Rfam [38]. The tRNAs were identified using the tRNAscan-SE (version 1.3.1) [39] with default parameters.

### 2.7. Evolution and Comparative Analysis

#### 2.7.1. Reconstruction of Gene Families

In addition to the *B. mucronatus* proteins, we also collected the protein sequences from 11 other species on Wormbase (https://www.wormbase.org/), including *Trichinella spiralis* (I)*, Ascaris suum* (III), *Brugia malayi* (III), *Meloidogyne hapla* (IV), *Steinernema carpocapsae* (IV), *B. xylophilus* (IV), *Panagrellus redivivus* (IV), *Strongyloides ratti* (IV), *Parastrongyloides trichosuri* (IV), *Haemonchus contortus* (V), *Caenorhabditis elegans* (V). After removing protein sequences shorter than 30 aa, or have frameshift, an All-to-All alignment of protein sequences was constructed using BLASTP (1e^−5^). OrthoFinder (version 2.2.7) [40] was then used to infer the orthologous genes and obtain the gene family based on the similarity of protein sequence. Café (version 3.0) [41] program was used to analyze expansion and contraction of gene families, in which the λ value was estimated by the program. The clade family number is defined as the shared family number of each species in each clade.

#### 2.7.2. Reconstruction of Phylogenetic Tree

Single-copy genes were extracted from OrthoFinder results and concatenated for reconstruction of the phylogenetic tree. The program Clustal Omega (Version 1.2.1) [42] was used for multiple sequence alignment. The gaps in the alignment were removed using the program trimAl with “–nogaps” (version 1.4) [43]. The optimal substitution model for amino acids was estimated by ProtTest (version 3.4.2) [44]. RAxML (version 8.0.24) [45] was used to reconstruct a phylogenetic tree based on maximum likelihood method using LG+I+G+F (LG model with +I: invariable sites; +G: gamma-distributed rates and +F: observed amino acid frequencies) substitution model suggested by ProtTest with 200 bootstrap replicates. *Trichinella spiralis* was used as the outgroup. Finally, iTOL [46] and Evolview [47] were used to visualize and annotated the phylogenetic tree.

#### 2.7.3. Collinear and Similarity Analysis of *B. mucronatus* and *B. xylophilus*

MCscan (https://github.com/tanghaibao/jcvi) was used to perform collinear analysis between *B. xylophilus* and *B. mucronatus*. LAST (Version 1021) [48] was used to perform the sequence alignments.

To assess the similarity of *B. mucronatus* and *B. xylophilus*, we compared the average mismatch rate of mapped reads when cross-mapping the sequencing reads of *B. mucronatus* and *B. xylophilus* to each other. BWA (Burrows-Wheeler Aligner) (Version 0.7.17-r1188) [49] was used to map sequencing reads to each reference genome. BAM (Binary Alignment Map) records that flagged as unmapped segments, secondary alignment, and supplementary alignment were filtered from mapping results using SAMtools [50]. The average mismatch rate was calculated by extracting “NM:i” tag from the BAM file to count the number mismatches and divide it by total number of mapped bases (sum of M, I, and D in the CIGAR string of BAM files). Meanwhile, the overall mapping rate was also calculated by SAMtools and compared between the two species.

### 2.8. Identification and Comparison of GPCR, Cellulase, and Pectin Genes of B. xylophilus and B. mucronatus

InterProScan results from gene annotation stage were used to identify GPCR, cellulase, and pectin genes in both *B. xylophilus* and *B. mucronatus*. As for GPCR genes, TMHMM and Pfam records were used to predict the transmembrane domain in the protein sequence. Proteins with seven transmembrane pattern and G Protein-Coupled Receptor domains were hypothetically identified as GPCR genes. As for cellulose and pectin genes, CAZy database [51] was used to search genes belongs to the carbohydrate active enzymes with BLASTP (1e^−5^).

The TPM (Transcripts Per Kilobase of exon model per Million mapped reads) of GPCRs for each gene was calculated using RSEM (Version 1.2.31) [52] with the default parameter. BINGO [53] was carried out for functional enrichment analysis.

## 3. Results and Discussion

### 3.1. Genome Assembly and Quality Assessment

**Figure 1:**
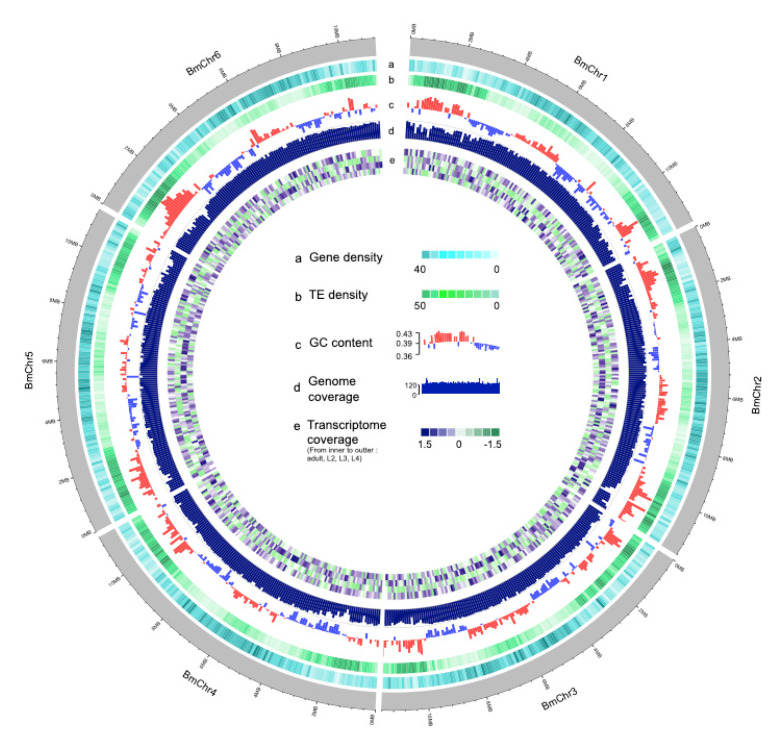
**Circos view of the *Bursaphelenchus mucronatus* genome**. The outer grey lanes depict circular representation of pseudo-molecules. The tracks indicate the following character with window size in 100 Kb: (**a**) the density of genes (darker color indicates more genes), (**b**) TE (Transposable element) density, (**c**) GC (Guanine and Cytosine) content, (**d**) genome coverage, (**e**) transcriptome coverage (from inner to outer indicate the different stage: adult, L2 (Second instar larvae), L3 (Third instar larvae), L4. (Fourth instar larvae). All values are normalized by Z-score).

**Figure 2:**
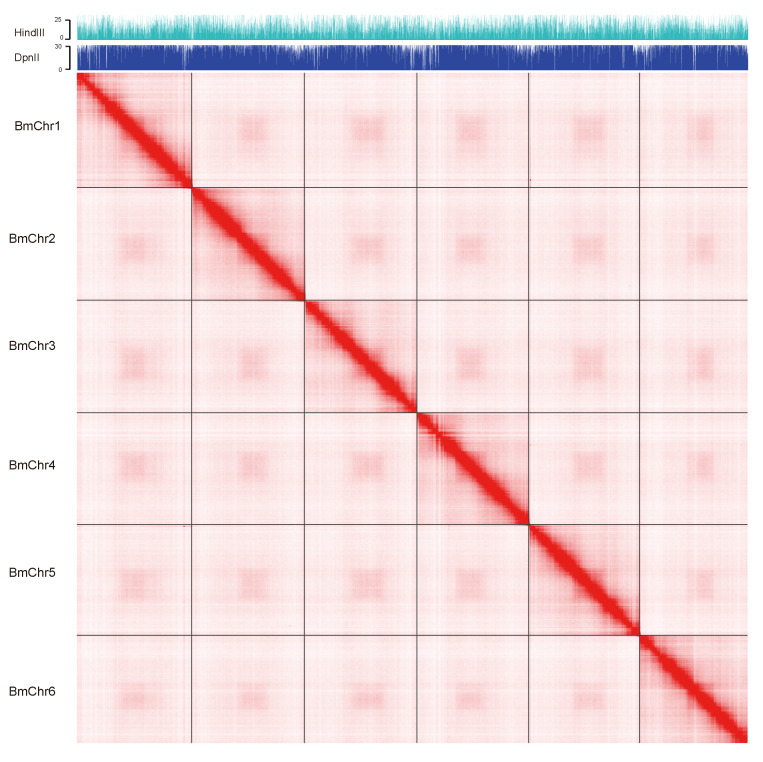
**Hi-C contact heatmap of pseudo-molecules.** The blocks represent signal associated with the two contact positions. All six blocks are evaluated by the genome coverage of Hi-C library of HindIII and DpnII.

Prior to the genome assembly, the genome size and heterozygosity rate were estimated using Illumina paired-end survey data. The estimated genome size based on 17-mer distribution was ~67.5 Mb (Figure S1), and the estimated heterozygosity rates was 0.257%. A total of 11 G single-molecule long reads (~160× based on estimated) were assembled using CANU. After pseudo-chromosome scaffolding using Hi-C data, we obtained a reference genome assembly of *B. mucronatus,* which consists of 72 super-scaffolds, including six pseudo-chromosomes (Figure 1 and Figure 2). The total size of the assembly was 73 Mb and scaffold N50 were 11.5 Mb (Table S6).

To assess the integrity and accuracy of the assembly, Illumina genome survey data and PacBio data were mapped to the assembly; the genome coverage was 97.11% and 95.68%, indicating that the assembly was relatively complete. The coverage map is showed in Figure S2. The number of complete and single-copy BUSCOs (Benchmarking Universal Single-Copy Orthologs) were 760 (77.4%) and 680 (69.2%), respectively, using protein and genomic models, which were comparable with *B. xylophilus* (Table S7). Meanwhile, the average transcriptomic-mapping rate was over 95% (Table S8), which also demonstrates that the protein-coding gene regions of the genome were also relatively complete.

### 3.2. Repeat and Non-Coding RNA Identification

The genome of *B. mucronatus* contained 21.74% repeat sequences (Table S9), similar to *B. xylophilus* [18]. We also identified 626 non-coding RNA (ncRNA) including 389 transfer RNAs (tRNAs), 183 ribosomal RNAs (rRNAs), 18 microRNAs (miRNAs), and 36 spliceosomal RNAs (Table S10).

### 3.3. Gene Annotation and Functional Analysis

A total of 13,696 non-redundant protein-coding genes were identified (Table S11). The average gene length is 3,564.61 bp and gene density is 187.48/Mb. The average exon length is 229.68 bp, exon GC (guanine and cytosine) content is 45.55%. We also performed functional analysis using mainstream public database and added annotations to the identified genes (Table S12). About 42% (5,761) and 32% (4,391) of genes were assigned to the GO (Gene Ontology) terms and KO (KEGG Orthology) identifiers, respectively. More than 60% of the genes were assigned to Pfam (11,317), NR (10,553) and UniProt (8,353). Among the gene set, 4,496 proteins contain a transmembrane domain from TMHMM annotation; these genes are candidates for receptor genes, such as GPCRs, which may sense external stimuli and chemical communication between populations [54,55].

### 3.4. Construction of Gene families

**Figure 3:**
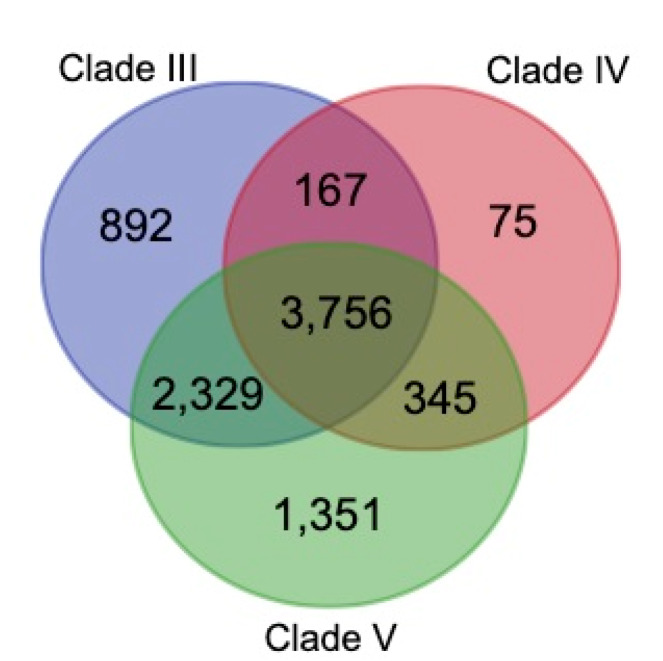
**Gene family numbers of three nematode clades.** The number of gene families for Clade IV shared 3923 with Clade III and 4101 with Clade V. The three clades shared 3756 gene families.

A total of 11 species were selected from Wormbase to perform gene family reconstruction using OrthoFinder (see method 2.7.1). Results showed that 252,937 protein sequences were classified into 14,951 families, of which *B. xylophilus* and *B. mucronatus* possessed 10,092 and 9,371 families, respectively. Results showed that Clade IV contains most of the species and possessed 4343 gene families, while Clade III and Clade V had 7,144 and 7,781 gene families, respectively. Clade IV shared 3,923 with Clade III and 4,101 with Clade V. These three clades shared 3756 gene families (Figure 3) that were enriched in the basic metabolic and biological process such as gene expression (*p* = 2.3e^−22^), RNA metabolic process (*p* = 7.5e^−13^), macromolecule biosynthetic process (*p* = 9.7e^−11^), structural molecule activity (*p* = 7.8e^−12^), ribonucleotide binding (*p* = 1.7e^−9^) and nucleotide binding (*p* = 3e^−9^) (Figure S3, Figure S4, Figure S5 and Table S13), which may be the fundamental functional genes for nematodes.

### 3.5. Genome Phylogeny of B. mucronatus

From OrthoFinder results, we collected 497 conserved single-copy genes in 12 species for reconstructing the phylogenetic tree. *T. spiralis* was selected as the outgroup to determine the root of the phylogenetic tree (Figure S6). The branch length of the topological tree indicated the expected value of substitutions per site. As shown in the phylogenetic tree, *B. xylophilus* and *B. mucronatus* were clustered together as neighborhood, which indicates that these two species were highly similar. The branch length of *B. xylophilus* and *B. mucronatus* were 0.0129 and 0.0202, respectively. This may indicate that mutations in the *B. mucronatus* may go a little bit further since last separation with *B. xylophilus.*

### 3.6. Dynamics of the Gene Families

**Figure 4:**
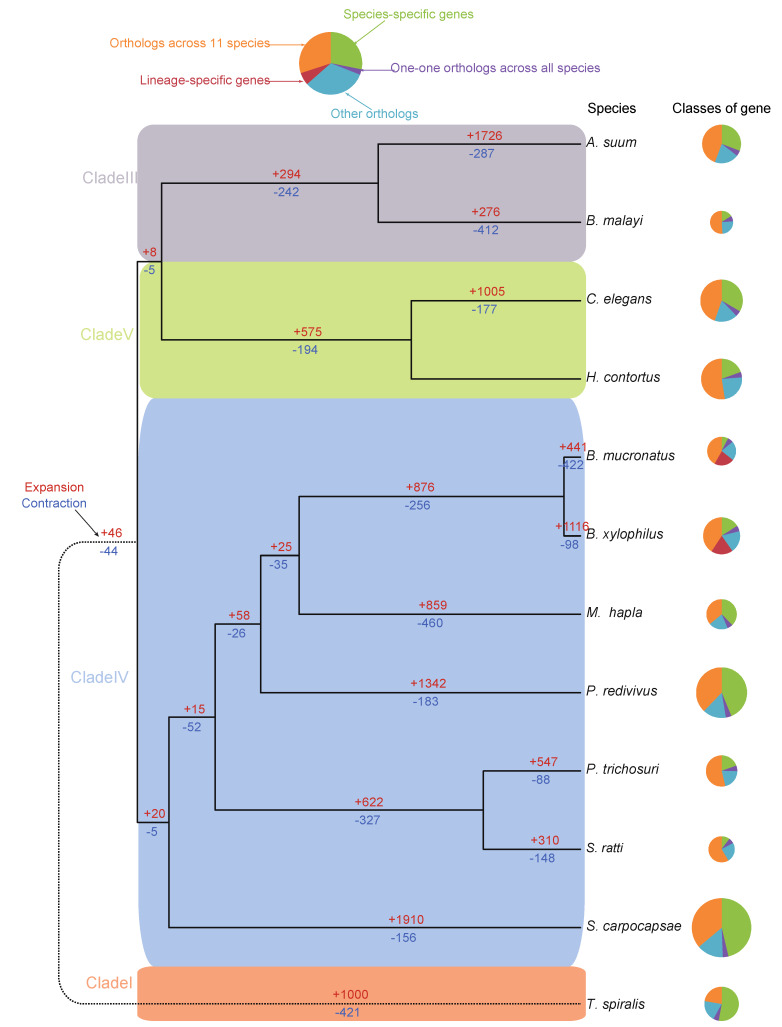
**Phylogeny and gene families of** ***B. mucronatus* and related species**. The phylogenetic relationship was inferred by RAxML, based on orthologous proteins of the 497 single-copy gene families. The circle size represents gene number, and the different colors indicate different gene types showed in the right top of the figure. The numbers above (red) and below (blue) indicate number of expansed and contracted gene families, respectively. The colors of the strips within the phylogenetic tree indicate different classes of the nematodes.

The dynamic change of gene families is a manifestation of species adaptive evolution at the gene level. In Figure 4**,** we showed the expansion/contraction of gene families on each branch of the species tree. Family expansions and contractions in Clade III and Clade V were 294 expansions vs. 242 contractions in Clade III and 575 expansions vs. 194 contractions in Clade V, respectively. As for Clade IV, each branch had a different number of gene family expansions vs. contractions. Looking at the numbers between *B. xylophilus* and *B. mucronatus*, *B. xylophilus* had 1,116 family expansions (4,462 genes) vs. 98 family contractions (215 genes), while *B. mucronatus* has 441 family expansions (1,417 genes) vs. 422 family contractions (1,184 genes). This may indicate that, in comparison with gene family enhancement in *B. xylophilus*, *B. mucronatus* are likely losing more species-specific genes, which might result in a relative lower ability to adopt to specific factor in environment than *B. xylophilus*.

Moreover, by looking at the functional enrichment of the gene families, we found that the family contractions of *B. mucronatus* were mainly enriched in transmembrane transport related processes such as transmembrane transport (Biological process, *p* = 6e^−5^), membrane (Cellular component, *p* = 5e^−6^), and transmembrane receptor activity (Molecular function, *p* = 1e^−4^), which may indicate *B. mucronatus* have weaker ability to respond to external stimuli (Figure S7, Figure S8, Figure S9, Table S14). Interestingly, in biological process, the G-protein coupled receptor (GPCR) protein signaling pathway was significantly enriched (*p* = 7e^−4^). Since GPCRs were related to the ability in the process of pheromone binding to the receptor or chemical communication, which is crucial with activities of nematodes, this might contribute to the displacement of *B. mucronatus* by *B. xylophilus* (Figure S7, Table S14).

The family expansions of *B. xylophilus* were mainly enriched in proteolysis (*p* = 2.2829e^−28^), peptidase activity (*p* = 2.0682e^−30^), and extracellular region (*p* = 3.0338e^−13^) (Figure S10, Figure S11, Figure S12, and Table S15). Interestingly, cellulase activity (*p* = 2e^−7^) showed significant enrichment in molecular function, in particular, the plant cell wall degradation (Figure S12, Table S15). We further annotated carbohydrate active enzymes (CAZymes) in both *B. mucronatus* and *B. xylophilus*. We found that GH45 family of cellulase was contracted in *B. mucronatus* (7 genes) while expanded in *B. xylophilus* (11 genes). Moreover, the PL3 family of pectinase showed a similar situation with GH4. In *B. mucronatus,* there were five genes, while in *B. xylophilus* there were 22 (Table S16). These results may indicate a considerable shift in the ability of plant cell wall degradation between *B. mucronatus* and *B. xylophilus* and, thus, may lead to weaker pathogenicity in *B. mucronatus*.

### 3.7. Genome Comparison of B. mucronatus and B. xylophilus

**Figure 5:**
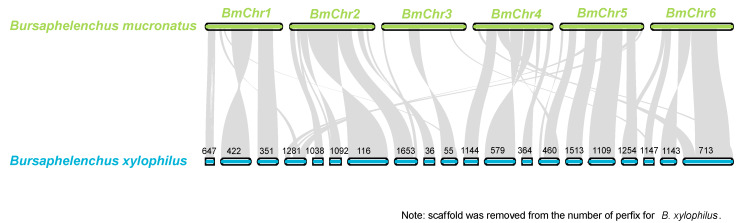
**Genome synteny between *B. mucronatus* and *B. xylophilus*.** Genomic alignments between *B. xylophilus* (Sequence length >1 Mb) and *B. mucronatus* showed high similarity of synteny. The alignments regions are marked with grey ribbons.

By analyzing the collinear and homology segments (Sequence length≥1 Mb) of the *B. mucronatus* and *B. xylophilus* using MCscan, we found that there are 183 homologous blocks between *B. xylophilus* and *B. mucronatus*, with a total length of about 44.86 Mb and a similarity of homologous segments of 88.71%. Genome structure showed high consensus of synteny between *B. xylophilus* and *B. mucronatus* (Figure 5), but the cross-mapping showed that the sequence varied greatly on the genome level and even greater on gene regions (Table S17). The synteny confirms that they originate from the same ancestor; however, their sequences have changed a lot before great structural variations taken place.

By analyzing the collinear and homology segments (Sequence length≥1 Mb) of the *B. mucronatus* and *B. xylophilus* using MCscan, we found that there are 183 homologous blocks between *B. xylophilus* and *B. mucronatus*, with a total length of about 44.86 Mb and a similarity of homologous segments of 88.71%. Genome structure showed high consensus of synteny between *B. xylophilus* and *B. mucronatus* (Figure 5), but the cross-mapping showed that the sequence varied greatly on the genome level and even greater on gene regions (Table S17). The synteny confirms that they originate from the same ancestor; however, their sequences have changed a lot before great structural variations taken place.

From the gene family results, we allocated all genes into three types, including species-specific orthologous clusters genes, un-clustered genes, and orphan genes as described in a previous study [56,57]. For *B. mucronatus*, there are 42 species-specific orthologous clusters genes, 241 un-clustered genes and 758 orphan genes, while the number in *B. xylophilus* is 194, 1,301, and 1,340. Functional analysis on species-specific orthologous clusters genes show that in *B. mucronatus* they mainly have kinase related function (PF00069|PF07714) (Table S18), while *B. xylophilus* have more than 10 varied conserved domains (Table S19). Interestingly, we found a 7TM GPCR, which belongs to the *srh* family in *B. xylophilus* species-specific orthologous clusters genes.

To investigate the possibility of gene variations which may contribute to the separation of the two species, we set three sequence identity cutoffs including (high, 0.5–0.75; medium, 0.33–0.50; and low, <0.33) to find out the number of ortholog pairs between the two species that have lower identity below the cutoff. We got 1,239, 354, and 104 genes in *B. mucronatus* and 1,633, 473, and 167 genes in *B. xylophilus* under each cutoff, respectively. Functional enrichment analysis results showed that in *B. xylophilus*, genes with medium identity were enriched in several stress responding processes including: responding to DNA damage stimulus stress, responding to stress, DNA repair, cellular response to stress, and cellular response to stimulus (Table S20). The gene function under the other two cutoffs of two species was mainly a basic biological process **(**Table S21**)**. This result indicates that orthologs of *B. xylophilus* in medium identity may contribute to its stronger environment adaption than *B. mucronatus*, which promotes its displacement of *B. mucronatus* during the invasion process.

### 3.8. GPCR Contractions May Be the Reason That B. mucronatus Was Displaced by B. xylophilus

We further extracted the GPCR genes of the two nematodes according to the Pfam and TMHMM results. In total, 551 GPCRs were found in *B. mucronatus* (Table S22 and Table S23) and 596 GPCRs were found in *B. xylophilus* (Table S24 and Table S25).

We further classified GPCRs based on Pfam annotation, including rhodopsin (104/106, *B. mucronatus*/*B. xylophilus*), secretin (6/5), srbc (2/2), srd (76/79), srh (82/98), sri (1/4), srj (3/3), srsx (42/44), srt (30/31), srv (5/7), srw (16/15), srx (14/16), str (117/125), class ab chemoreceptor (53/61). This number distribution (Figure S13) indicates that some type of GPCRs may be losing diversity in *B. mucronatus*, which may indicate overall reduced sensitivity in response to chemical stimulation. Combined with gene family results, we found that the number of single-copy genes, *srn-1*(srh family), whose function in *C. elegans* is amphid sensory neurons [58] is much higher in *B. xylophilus* (22 genes) than in *B. mucronatus* (13 genes). This result may indicate that *B. xylophilus* has better migration ability than *B. mucronatus*, which confirms the previous phenotypic experiments [59]. Meanwhile, *npr-1* (rhodopsin family)*,* the gene function of feeding behavior, aerotaxis, thermal avoidance, ethanol tolerance, innate immunity in *C. elegans* [60], has single copy in *B. xylophilus* and no ortholog in *B. mucronatus.* The *npr-5* (rhodopsin family)*,* whose function is related to fat storage and dauer formation in *C. elegans* [60], has the same situation as *npr-1*. The loss of the npr genes may lead to weaker stress resistance of *B. mucronatus*. When the environment is poor and the food is lacking, this may lead *B. mucronatus* to enter the dauer formation stage at a much slower speed, after the quickly-responding *B. xylophilus* and the weaker fat synthesis and storage capacity will lead to death. However, *B. xylophilus* will quickly enter the dauer formation stage, the fat particles in the body increase in order to endure the stress. Furthermore, we find that the *daf-38* (rhodopsin family) that was mediating ascaroside perception in *C. elegans* has five copies in *B. xylophilus*, and two copies in *B. mucronatus,* which may result in a weaker ability to conduct chemical communications within *B. mucronatus* populations.

In a recent study, we found that displacement between *B. xylophilus* and *B. mucronatus* was facilitated by ascarosides [61]. The two nematode species reacted differently to the same concentration of ascarosides. Low concentration of ascarosides could promote the growth and the length of the female body of *B. xylophilus,* while the same pheromone had no effect on the *B. mucronatus* [61].

We also checked the RNA-seq data for any evidence to support this hypothesis. Because of no biological replicate and potential wild sampling bias, we simply used mixed age RNA-seq of *B. xylophilus* as well as *B. mucronatus* to compare their overall expression levels. The results showed that the overall expression level of all GPCRs of *B. xylophilus* is relatively higher than *B. mucronatus* (Table S26, Table S27, Figure S14). This indicates that during the development stages, in addition to the copy number expansion, the expression level may also contribute to a higher quantity of GPCRs in *B. xylophilus*, which may lead to higher sensitivity to external pheromones stimuli in *B. xylophilus*.

## 4. Conclusions

In this study, we introduced the first chromosome-level *B. mucronatus* genome using PacBio and Hi-C technology, which brings a valuable genomic resource for molecular and evolutionary studies of *B. mucronatus*, especially a fundamental resource for studying the competitive displacement by the pinewood nematode *B. xylophilus*. With this new resource, previous comparative studies that focused on biological characteristics between *B. mucronatus* and *B. xylophilus* [7,11,12,13] could be extended to the genomic and molecular level in the near future. Moreover, the comparative genomic analysis would also finally decipher the code behind the competitive displacement between these two nematodes, which is the key role to understand and control the pathogenicity of pine wilt diseases.

In this study, we identified ‘Species-identity-specific genes’ from the orthologs under different identity cutoffs. Functional enrichment analysis results showed that medium identity of *B. xylophilus* genes are enriched in several stress responding process. Meanwhile, species-identity-specific genes of the *B. mucronatus* are mainly enriched in fundamental protein biological processes. This result may indicate that the sequence difference in orthologues may contribute to the speciation process.

As shown in the phylogenetic tree, *B. xylophilus* and *B. mucronatus* were clustered together as neighborhood, which indicates that these two species were highly similar. However, we noticed that the phylogenetic tree showed slightly different structure from previous nematode study [62]. This may be due to the different datasets used for tree inferences. In prior to the whole genome, mitochondrial genes were often used [63]. In our study, all coding genes were used for the tree inference. We also checked and confirmed the similar situation in some other studies [64,65]. We cannot justify which one is more correct, but this at least indicates different mutation rates on the mitochondrion and the whole genome.

The gene family contraction of *B. mucronatus* and the gene family expansion of *B. xylophilus* showed that some type of GPCRs may be missing or losing diversity in *B. mucronatus*, which may result in a weaker ability of *B. mucronatus* in the process of pheromone binding to the receptor or chemical communication and pathogenicity compared with *B. xylophilus*. In previous studies, it was found that *B. mucronatus* was the weaker contender in the competitive displacement by *B. xylophilus* [12,66]. From the RNA-seq results, it is implied that higher sensitivity of GPCRs in *B. xylophilus* makes it easier for the signal transduction to reach its saturation. This may also trigger the feedback regulation on expression of GPCRs once saturated [67], which eventually reduce the expression levels of GPCRs and, thus, lower its sensitivity to external pheromones stimuli. On the opposite, the lower expression levels of GPCRs in *B. mucronatus* may lower its sensitivity and raise the threshold of ascarosides, thus, making it harder to response to external stimuli, which may result in a null effect on fecundity or even suppression. This may indicate that the gene family expansion and gene regulation may be responsible for the higher baseline expression level of GPCRs in *B. xylophilus* than in *B. mucronatus*. Overall, the enhanced chemical sensing of ascarosides in *B. xylophilus* and diminished ability in *B. mucronatus*, which result in physiological changes that favor the enhanced fecundity and length growth of *B. xylophilus*, may be an explanation, and indicate that the displacement of *B. mucronatus* could be a chemical communications-driven procedure.

Overall, we introduced a high quality reference genome for *B. mucronatus*, compared it with the released reference genome of *B. xylophilus* [18], and discussed the phenomenon of displacement from the view of gene families, functions, and expressions. In the process of invasion by alien species, chemical communication plays a critical role in the displacement between invasive and indigenous species [68]. In the *B. xylophilus*–*B. mucronatus* system, we believe that differences in the contraction (*B. mucronatus*) and expansion (*B. xylophilus*) of the GPCR families may be related to the different responses to ascarosides, which is one of the key factors in their apparent displacement. With these changes, *B. mucronatus* may be less sensitive to ascarosides than *B. xylophilus*. Meanwhile, our research helps to reveal that the internal essence of the displacement between these two species is likely highly facilitated by chemical communication, which potentially provides new perspectives for pine wilt disease control, and mitigating the negative impacts of such biological invasion.

## Supplementary Materials

The following are available online at https://www.mdpi.com/2073-4425/11/5/570/s1, Figure S1: GenomeScope k-mer frequency and coverage profiles of genome survey data (k = 17). Figure S2: Hi-C contact heatmap with PacBio and Illumina data evaluated of pseudo-molecules. The largest scale of PacBio data and Illumina data are 120 and 150, respectively. Figure S3: Biological process enrichment of shared gene family in three Clades. The annotation background is whole gene GO annotation result from BINGO. Figure S4: Cellular component enrichment of shared gene family in three Clades. The annotation background is whole gene GO annotation result from BINGO. Figure S5: Molecular function enrichment of shared gene family in three Clades. The annotation background is whole gene GO annotation result from BINGO. Figure S6: Phylogenetic tree of *B. mucronatus* and related species. The phylogeny was constructed based on the protein sequences of the 497 single-copy genes. The values above the branches indicate the branch (solid line) lengths. Figure S7: Biological process enrichment of the contracted genes families of *B. mucronatus*. Figure S8: Cellular component enrichment of the contracted genes families of *B. mucronatus*. Figure S9: Molecular function enrichment of the contracted genes families of *B. mucronatus*. Figure S10: Biological process enrichment of the expanded families of *B. xylophilus*. Figure S11: Cellular component enrichment of the expanded families of *B. xylophilus*. Figure S12: Molecular function enrichment of the expanded families of *B. xylophilus*. Figure S13: Distribution of GPCR subfamily in *B. xylophilus* and *B. mucronatus*. Figure S14: Boxplot of GPCRs expression in *B. xylophilus* and *B. mucronatus*. The average expression of *B. xylophilus* is higher than *B. mucronatus*. Table S1: Statistics of genome survey data of *B. mucronatus*. The 4-peak model was used to estimate the heterozygosity, genome haploid length (bp), genome repeat length (bp), genome unique length (bp) and read error rate, which was converged in 4 rounds. Table S2: Statistics of PacBio sequencing data of *B. mucronatus*. Table S3: Statistics of different parameter combination of Canu assembly. Spec.file is the specific parameter in each round. Repeat-dove reads are short for dovetail, where the ends of reads align fully. Repeat-cont are short for contained, where a read aligns fully within another read. Uniq-repeat-cont reads are fully contained in other read. Uniq-repeat-dove reads are not fully contained in other read, which was used to form initial contigs. Span-repeat is the spanning repeat reads. Uniq-anchor reads are used for anchoring contig. Contigs are the initial contig of Canu assembly. Consensus is the final contig assembly of Canu. Table S4: Statistics of *B. mucronatus* contig feature. Table S5: Statistics of Hi-C sequencing data of *B. mucronatus*. Table S6: Statistics of *B. mucronatus* genome feature. Table S7: The result of BUSCO. Table S8: The mapping rate of RNA-seq data. Table S9: Summary of repeat elements identified in the *B. mucronatus* genome. LINEs: long interspersed elements, SINEs: short interspersed elements, LTR elements: long terminal repeat, DNA elements: DNA type repeat. Table S10: Non-coding RNAs in the *B. mucronatus* genome. Table S11: Statistics of *B. mucronatus* gene annotation. Table S12: Function annotation of *B. mucronatus*. Table S13: Functional enrichment of shared gene family in three Clades. Table S14: Functional enrichment of contraction family of *B. mucronatus*. Table S15: Functional enrichment of expansion family of *B. xylophilus*. Table S16: Statistics of cellulase family of *B. xylophilus* and *B. mucronatus*. Table S17: Statistics of average error rate of cross-mapping between *B. mucronatus* and *B. xylophilus*. Table S18: Functional annotation of species-specific cluster genes of *B. mucronatus*. Table S19: Functional annotation of species-specific cluster genes of *B. xylophilus*. Table S20: Functional enrichment of median identity ortholog genes of *B. xylophilus*. Table S21: Functional enrichment of different identity cutoff of ortholog genes in *B. xylophilus* and *B. mucronatus*. Table S22: Pfam annotation of GPCR genes of *B. mucronatus*. Table S23: TMHMM annotation of GPCR genes of *B. mucronatus*. Table S24: Pfam annotation of GPCR genes of *B. xylophilus*. Table S25: TMHMM annotation of GPCR genes of *B. xylophilus*. Table S26: The expression of GPCRs in *B. xylophilus*. Table S27: The expression of GPCRs in *B. mucronatus*.
