# Supplementary figures and images for: A Reference Genome of Bursaphelenchus mucronatus Provides New Resources for Revealing Its Displacement by Pinewood Nematode
Source: Genes (Basel). 2020 May 19;11(5):570. doi: 10.3390/genes11050570 (PMC7288286; doi:10.3390/genes11050570)

Figure S8

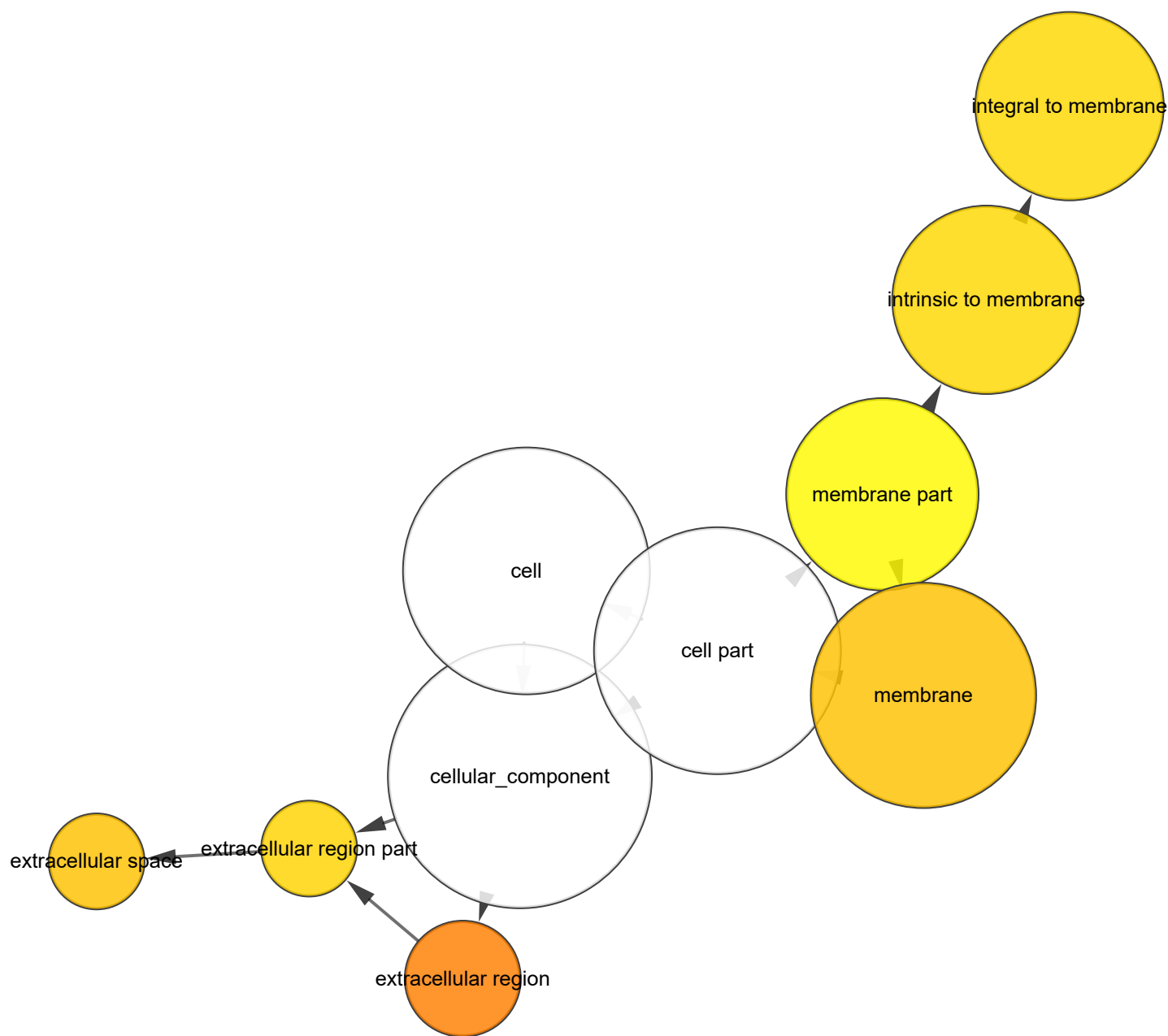

Supplement: Supplementary file 1 [file genes-11-00570-s001.zip › genes-746709-supplementary/Supp-Figure/Figure S8.pdf]

## Figure S9

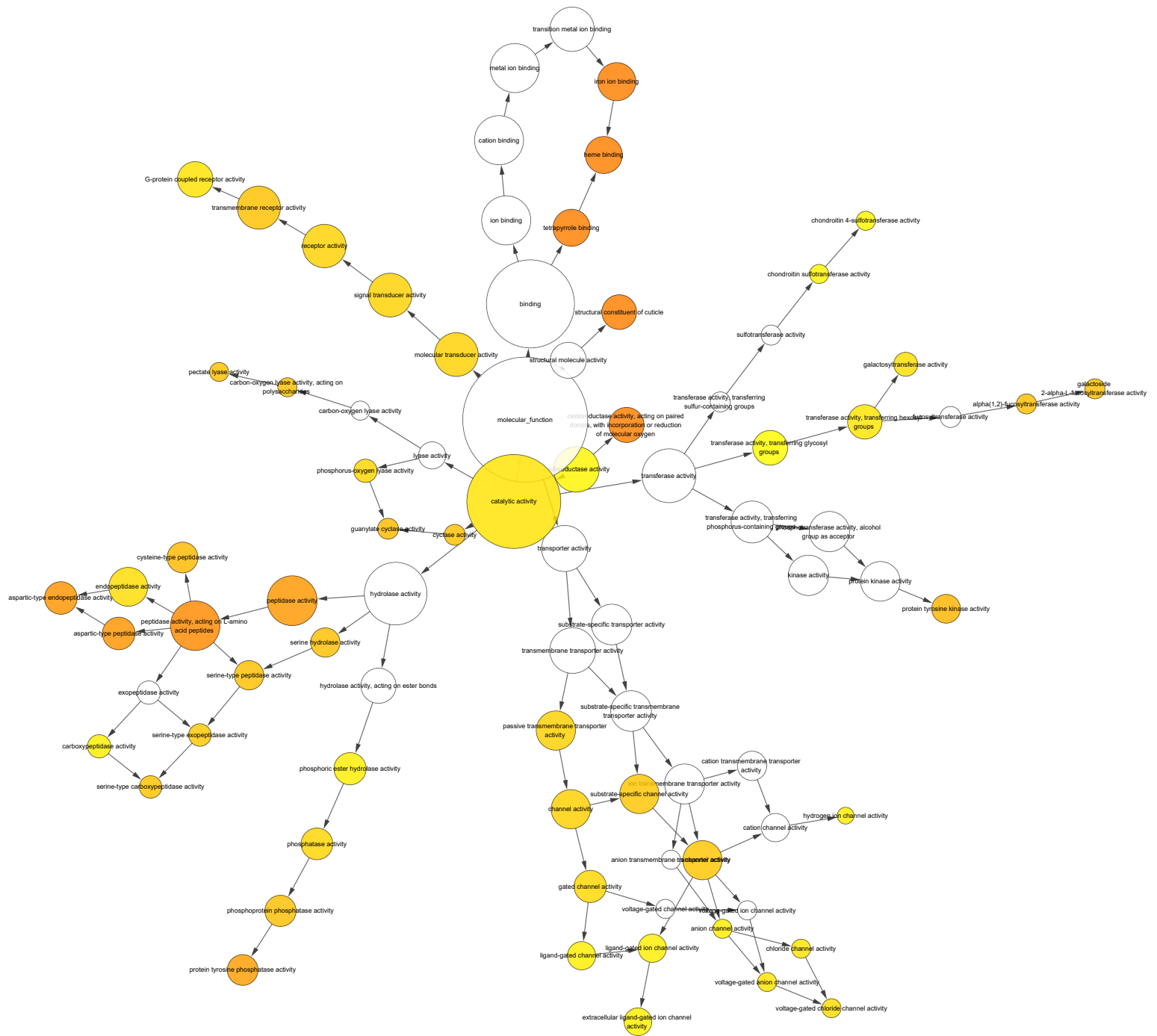

Supplement: Supplementary file 1 [file genes-11-00570-s001.zip › genes-746709-supplementary/Supp-Figure/Figure S9.pdf]

Figure S24

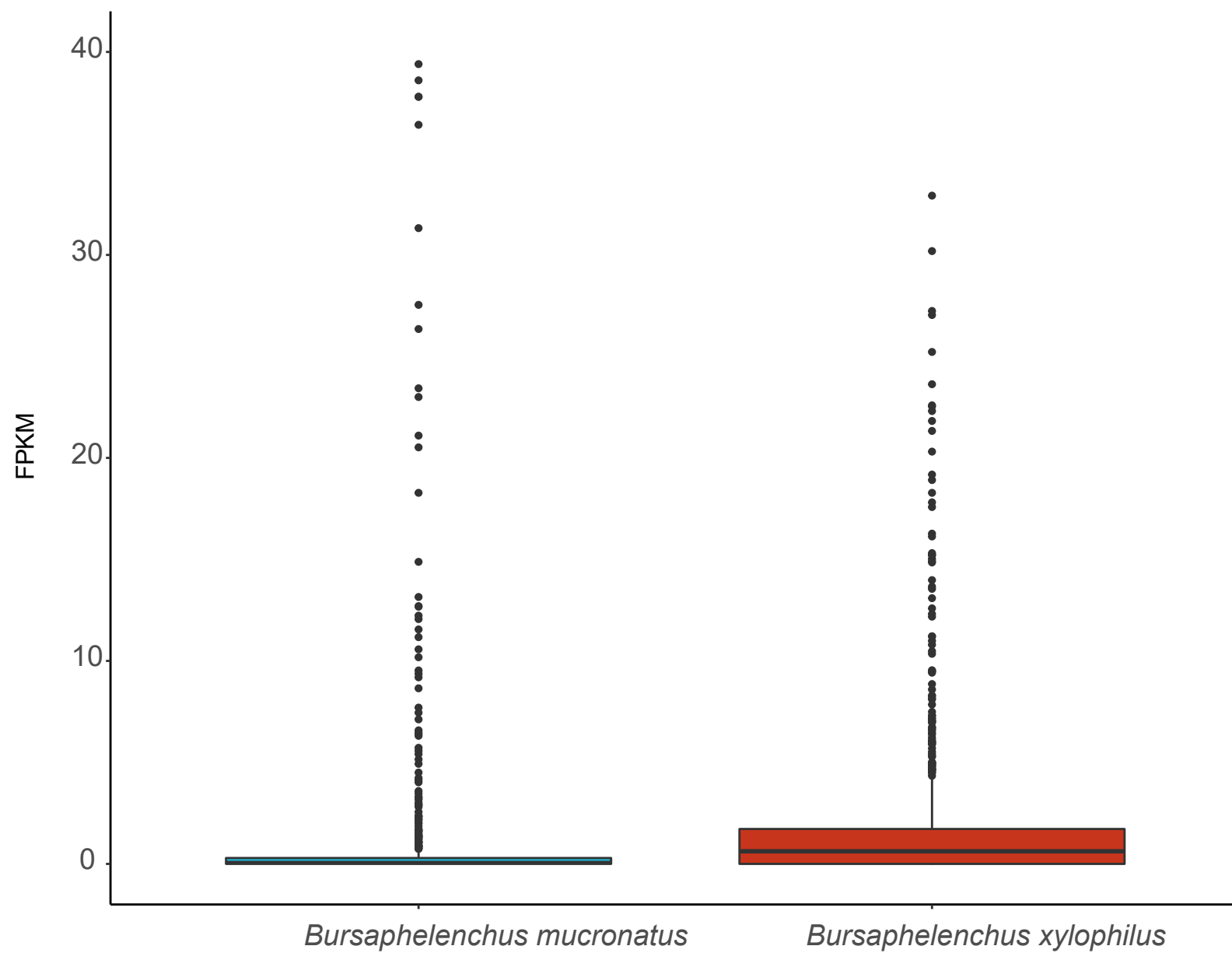

Supplement: Supplementary file 1 [file genes-11-00570-s001.zip › genes-746709-supplementary/Supp-Figure/Figure S14.pdf]

Figure S11

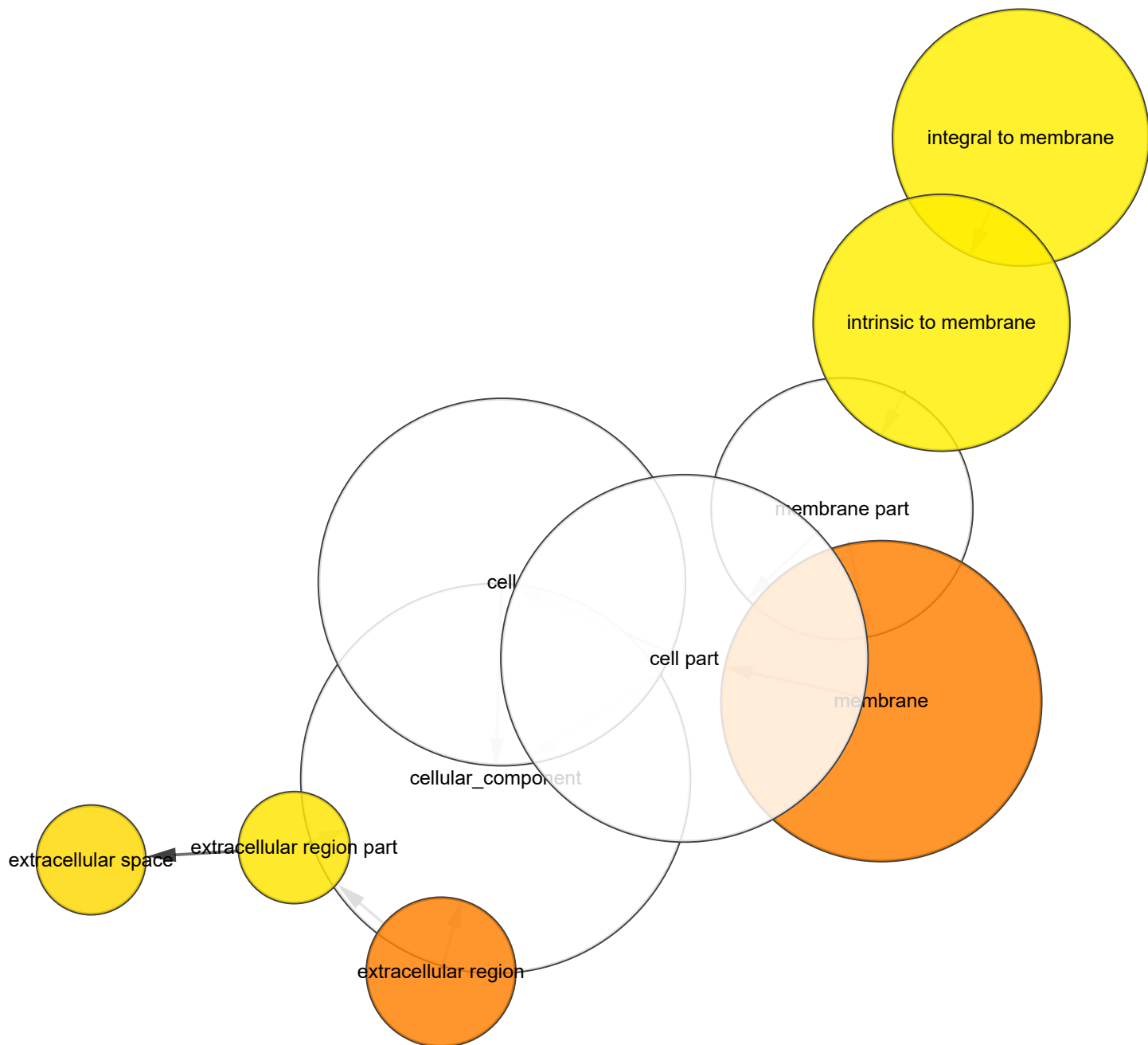

Supplement: Supplementary file 1 [file genes-11-00570-s001.zip › genes-746709-supplementary/Supp-Figure/Figure S11.pdf]

Figure S10

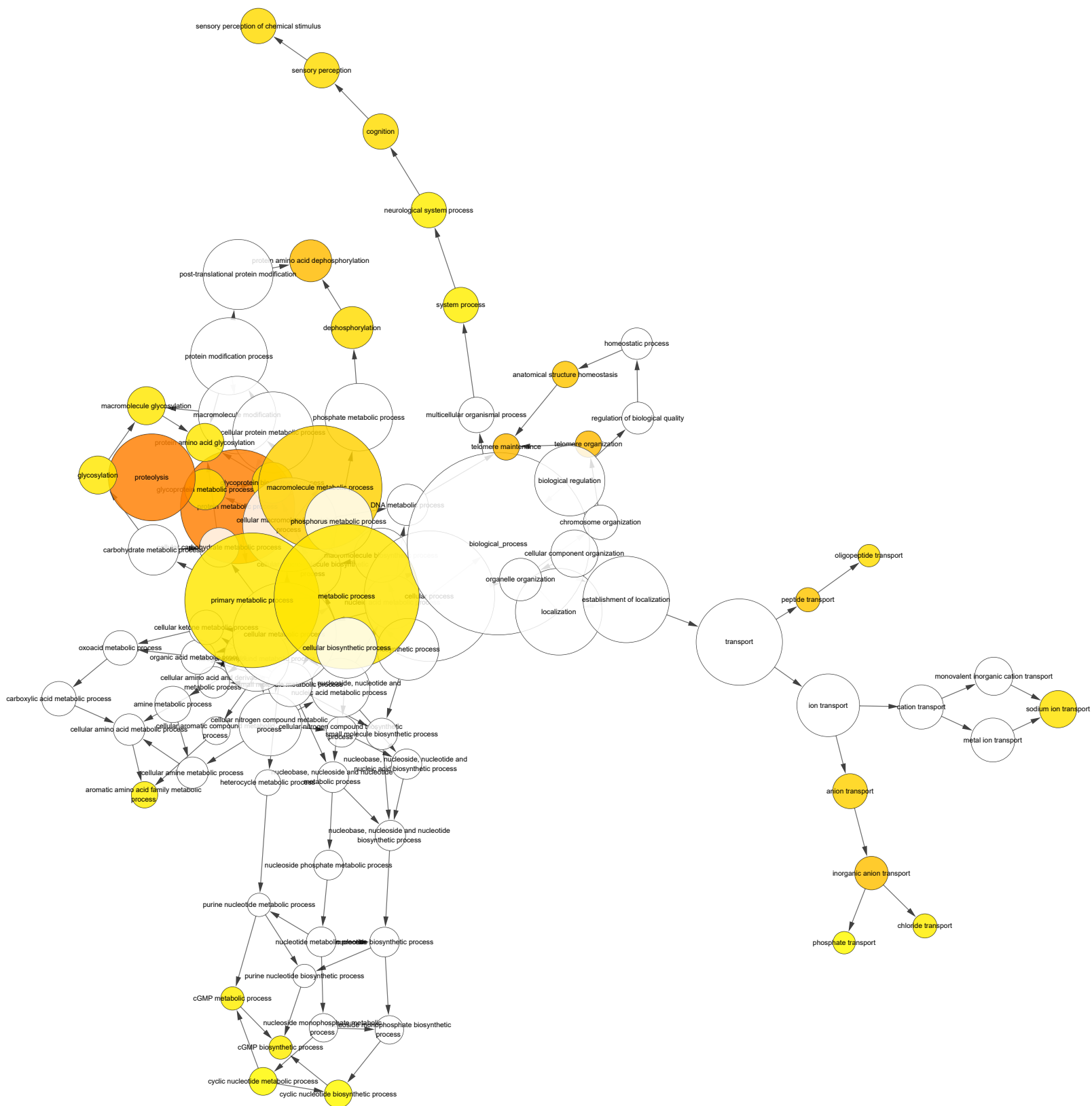

Supplement: Supplementary file 1 [file genes-11-00570-s001.zip › genes-746709-supplementary/Supp-Figure/Figure S10.pdf]

Figure S12

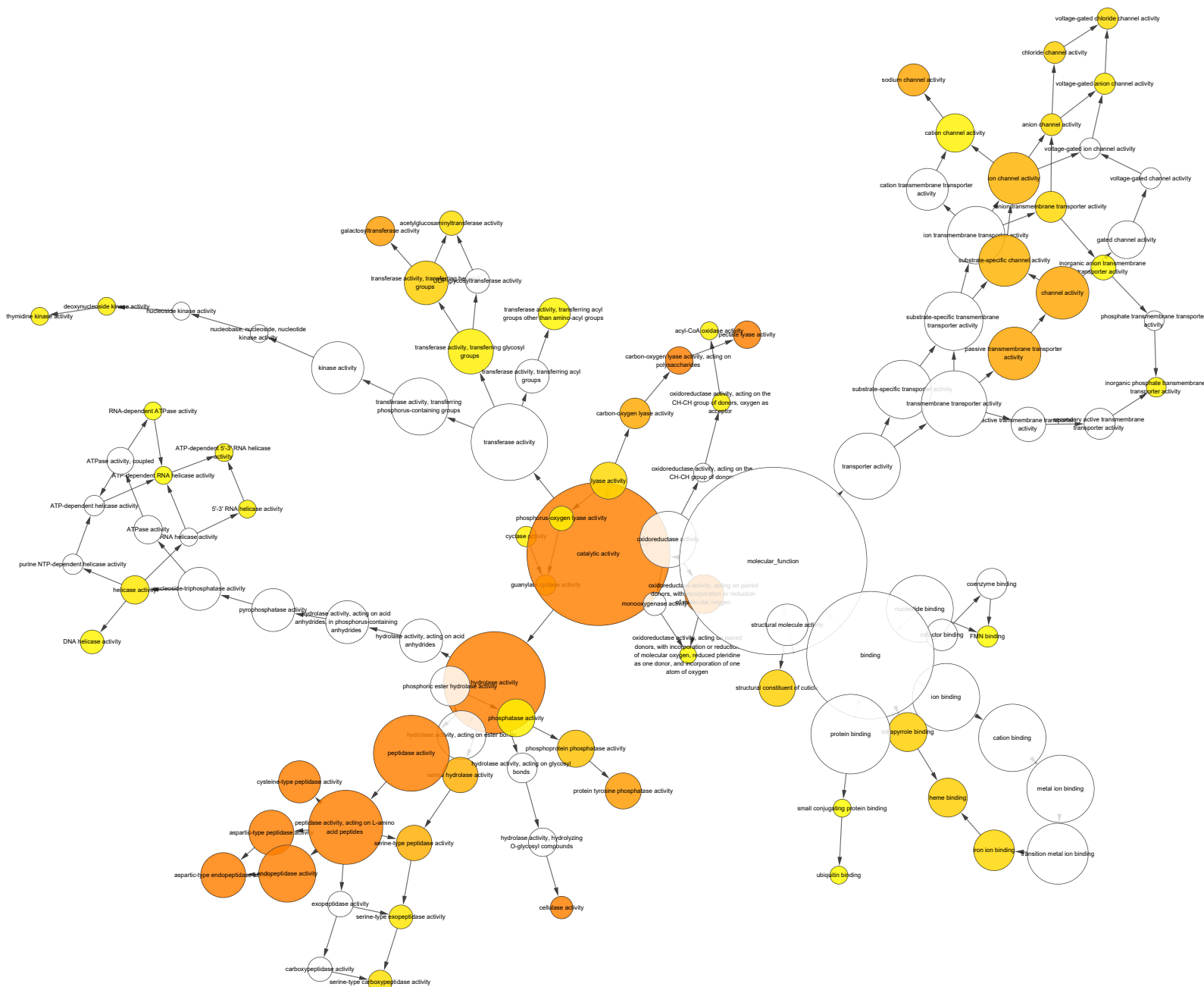

Supplement: Supplementary file 1 [file genes-11-00570-s001.zip › genes-746709-supplementary/Supp-Figure/Figure S12.pdf]

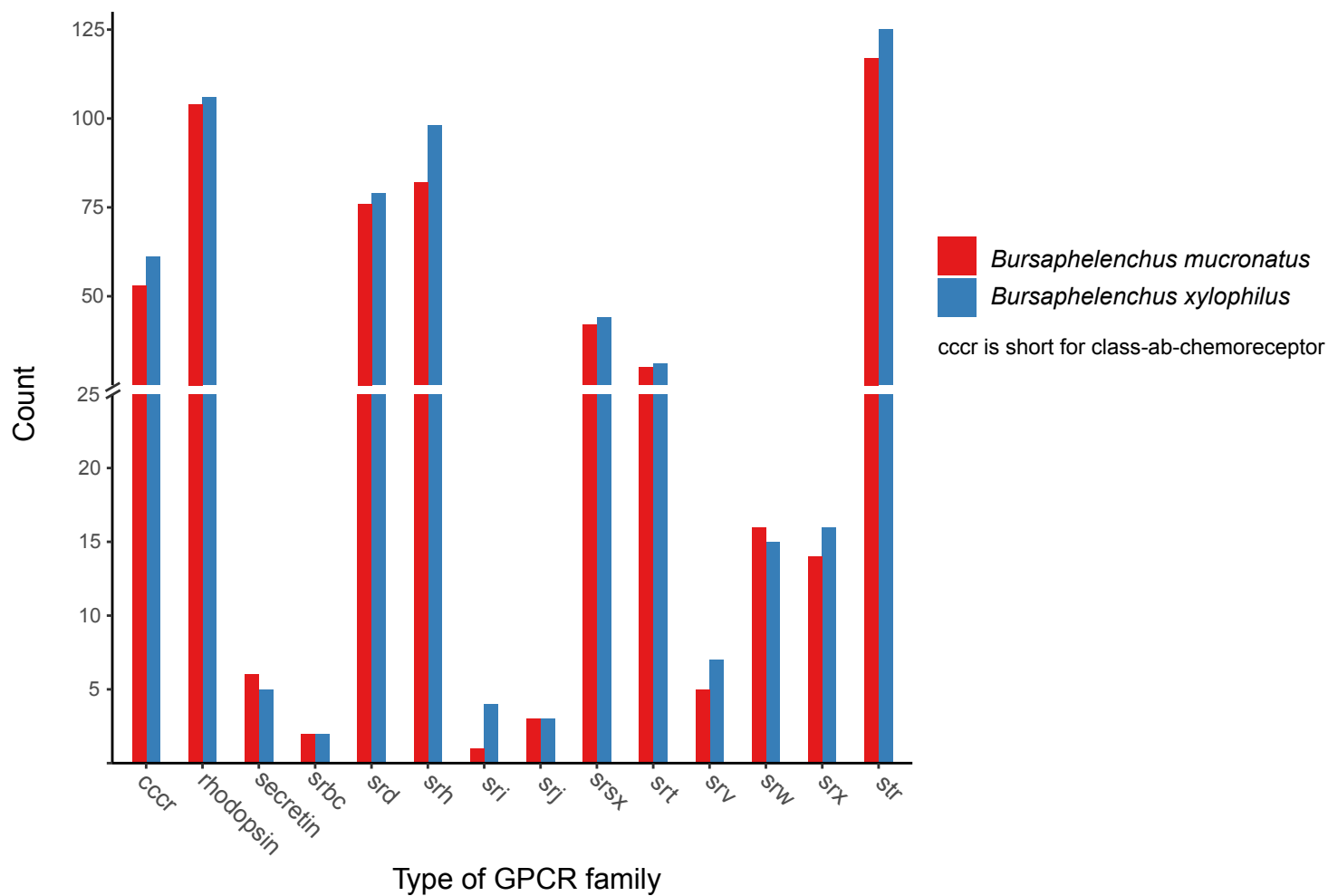

Supplement: Supplementary file 1 [file genes-11-00570-s001.zip › genes-746709-supplementary/Supp-Figure/Figure S13.pdf]

Figure S1

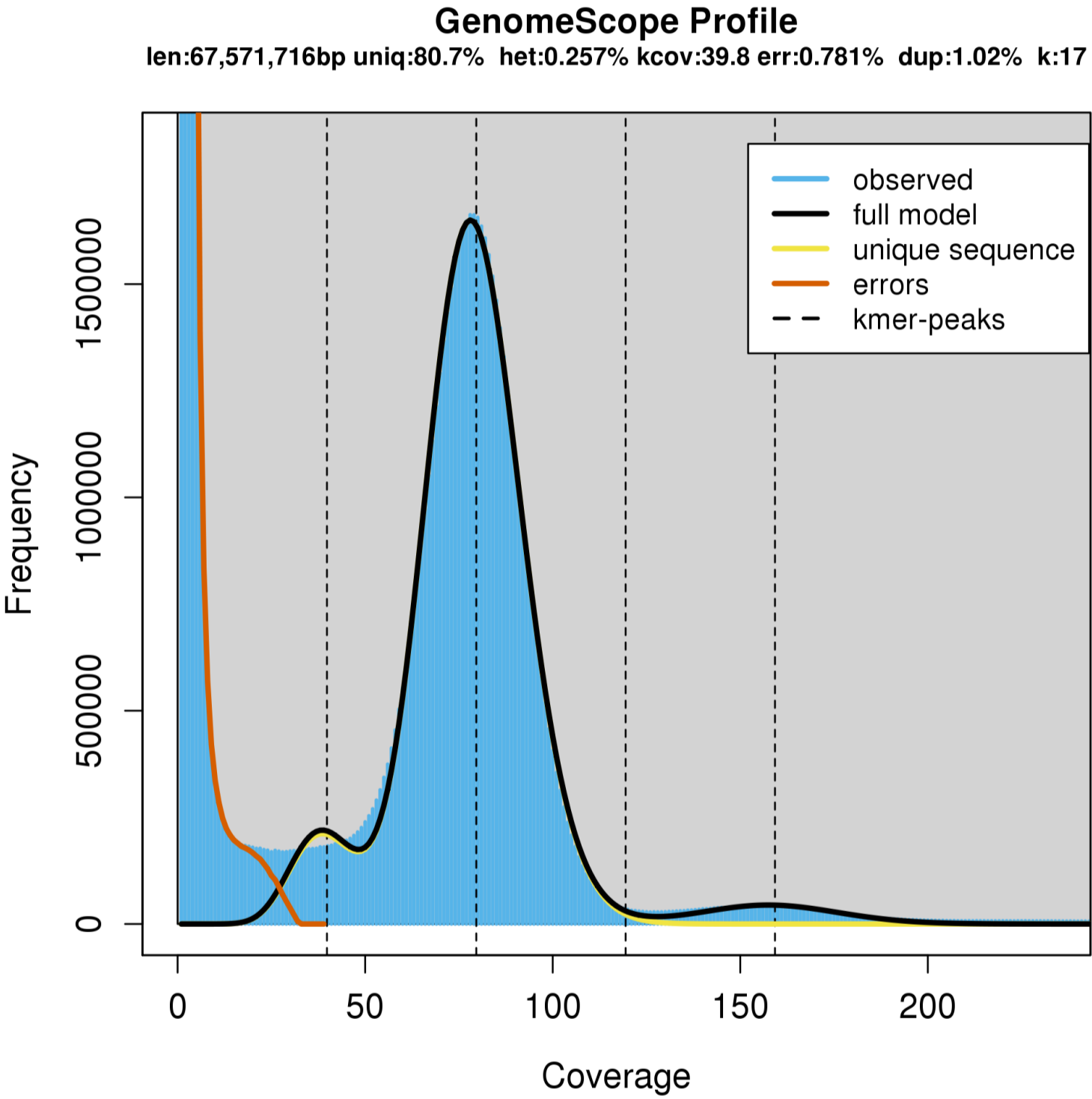

Supplement: Supplementary file 1 [file genes-11-00570-s001.zip › genes-746709-supplementary/Supp-Figure/Figure S1.pdf]

Figure S2

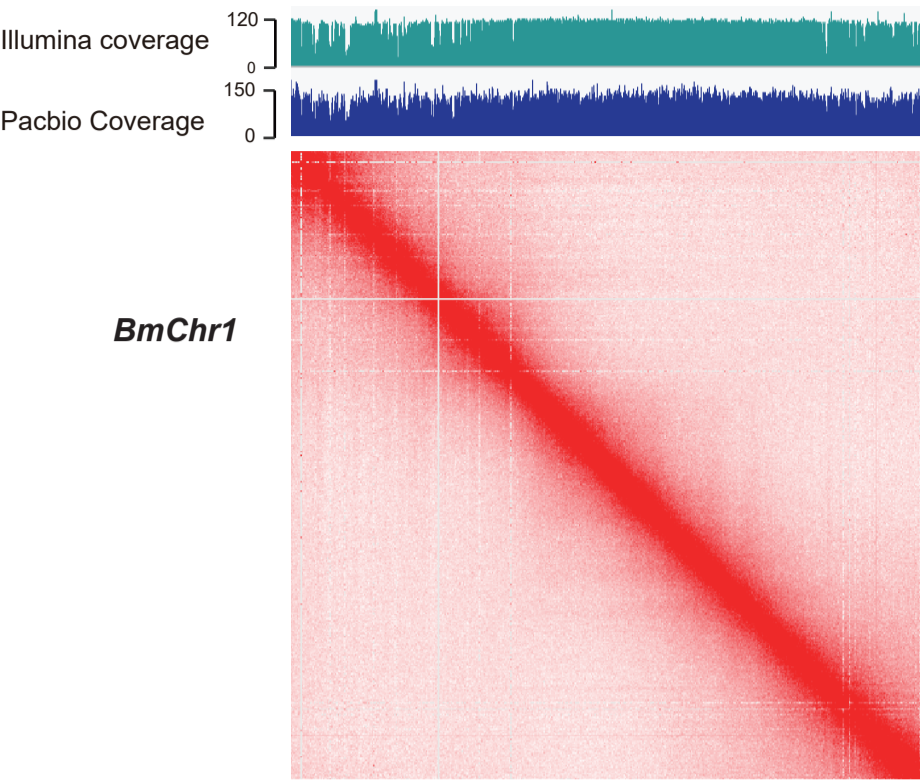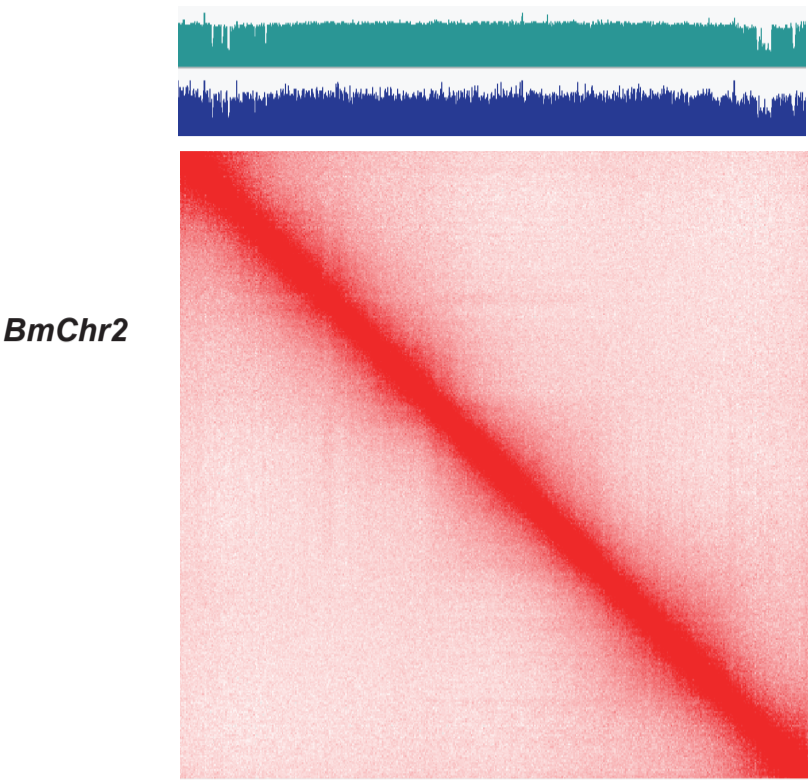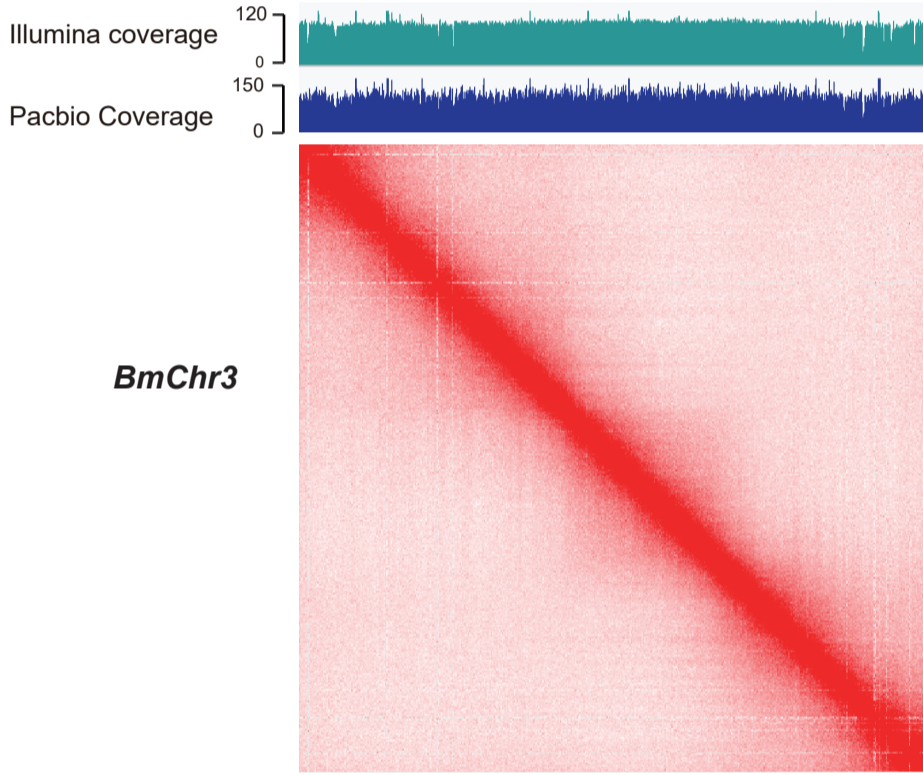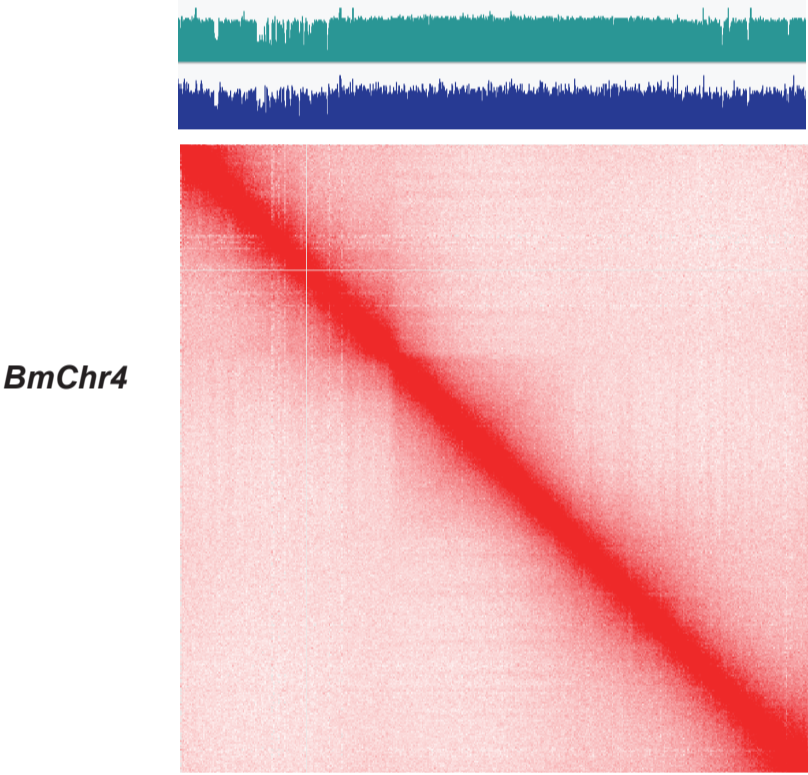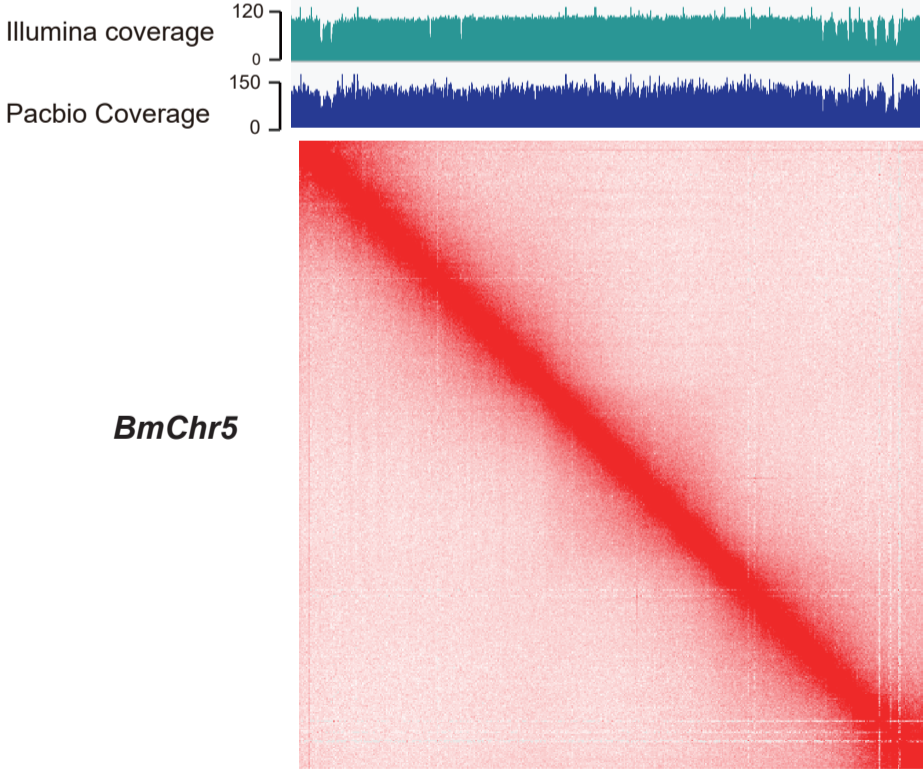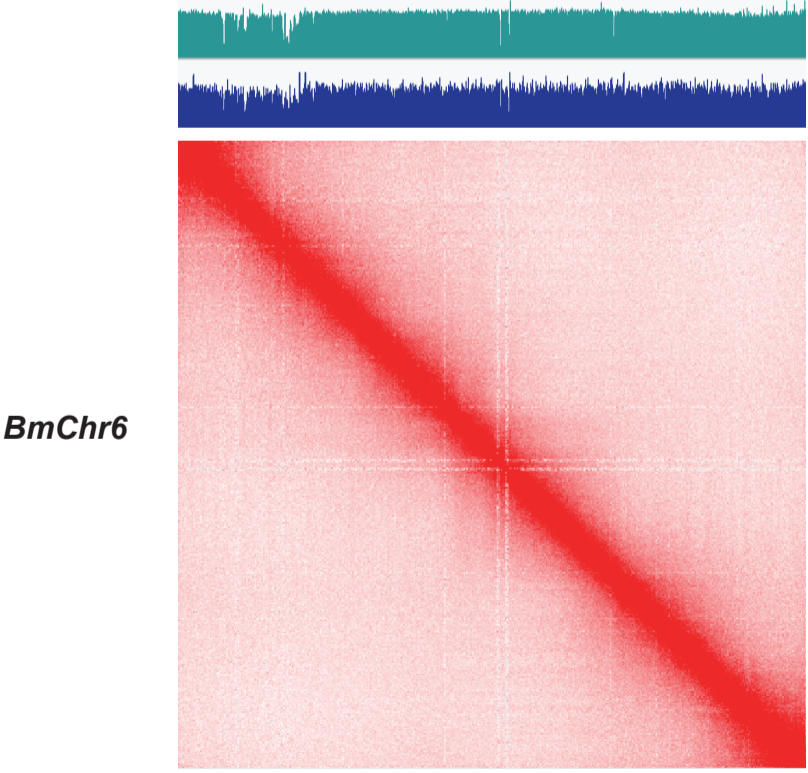

Supplement: Supplementary file 1 [file genes-11-00570-s001.zip › genes-746709-supplementary/Supp-Figure/Figure S2.pdf]

## Figure S3

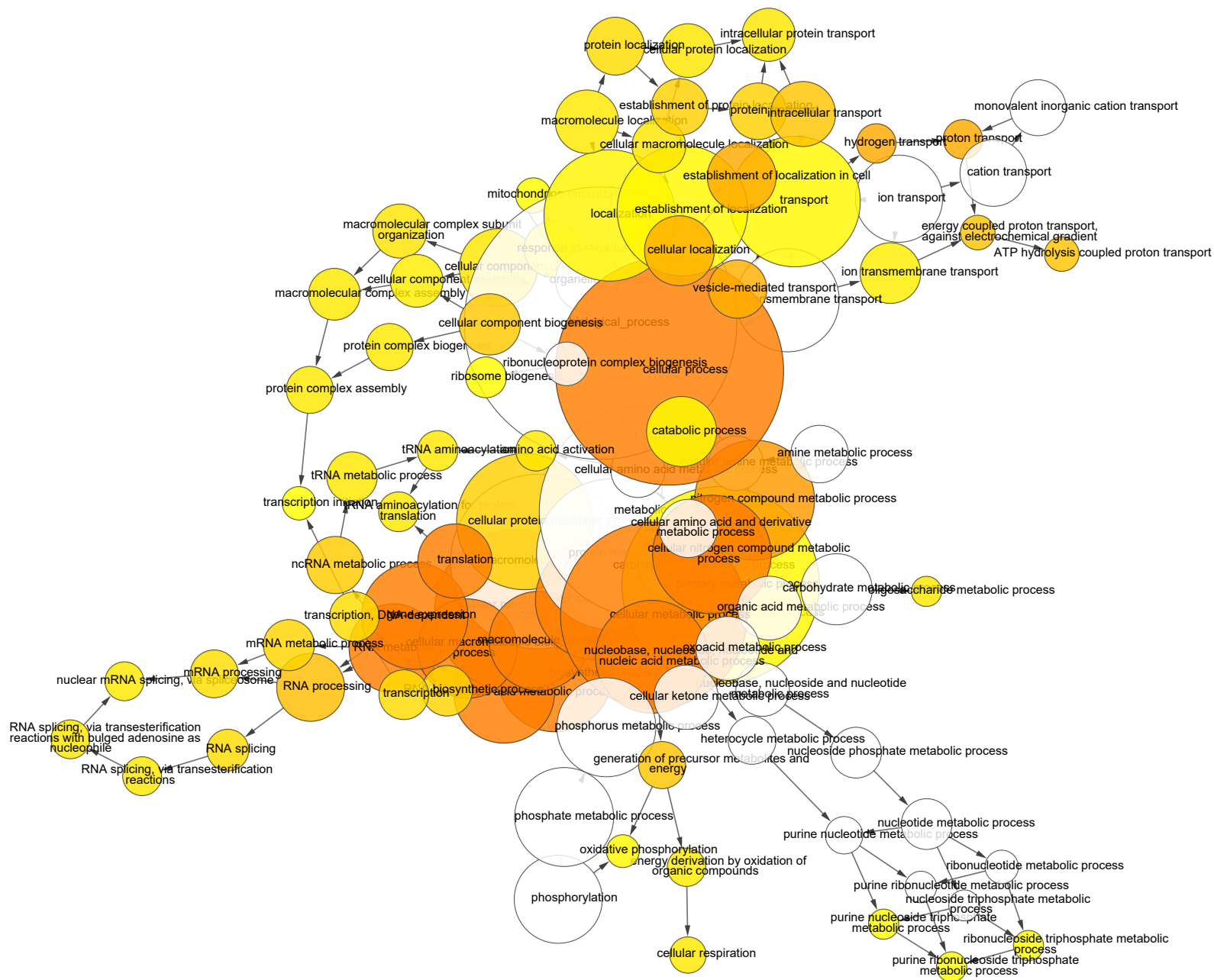

Supplement: Supplementary file 1 [file genes-11-00570-s001.zip › genes-746709-supplementary/Supp-Figure/Figure S3.pdf]

Figure S7

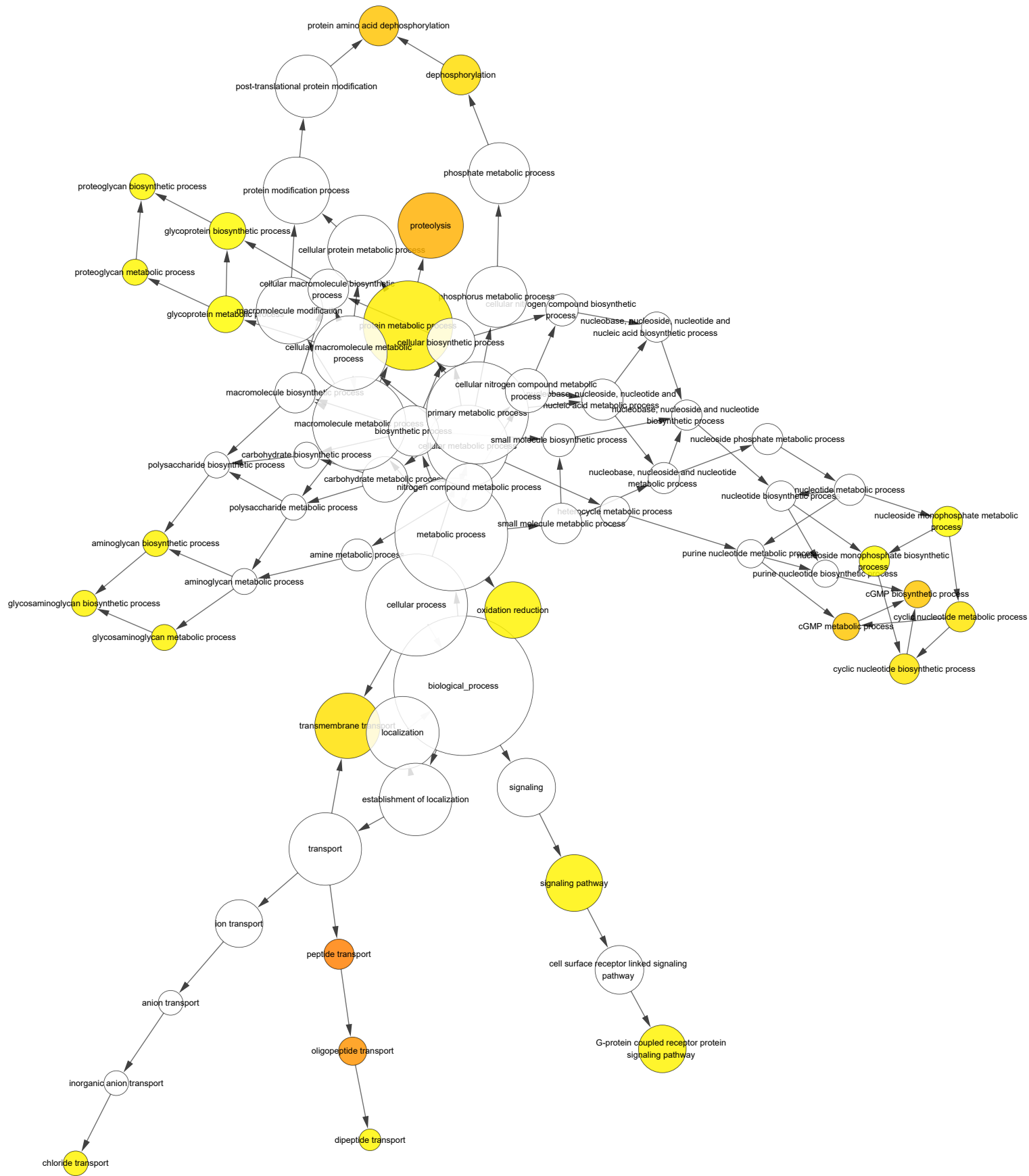

Supplement: Supplementary file 1 [file genes-11-00570-s001.zip › genes-746709-supplementary/Supp-Figure/Figure S7.pdf]

Figure S6

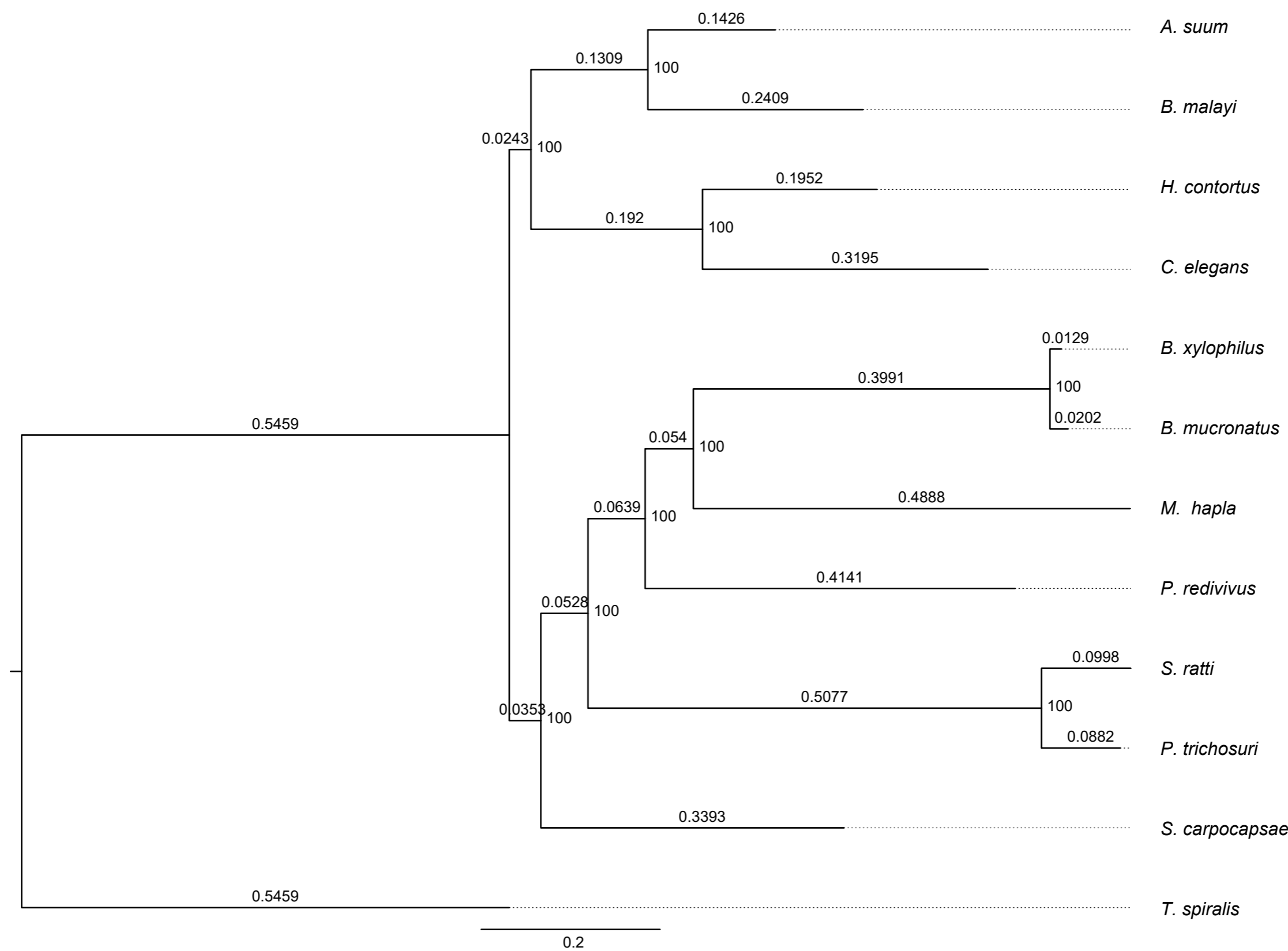

Supplement: Supplementary file 1 [file genes-11-00570-s001.zip › genes-746709-supplementary/Supp-Figure/Figure S6.pdf]

Figure S4

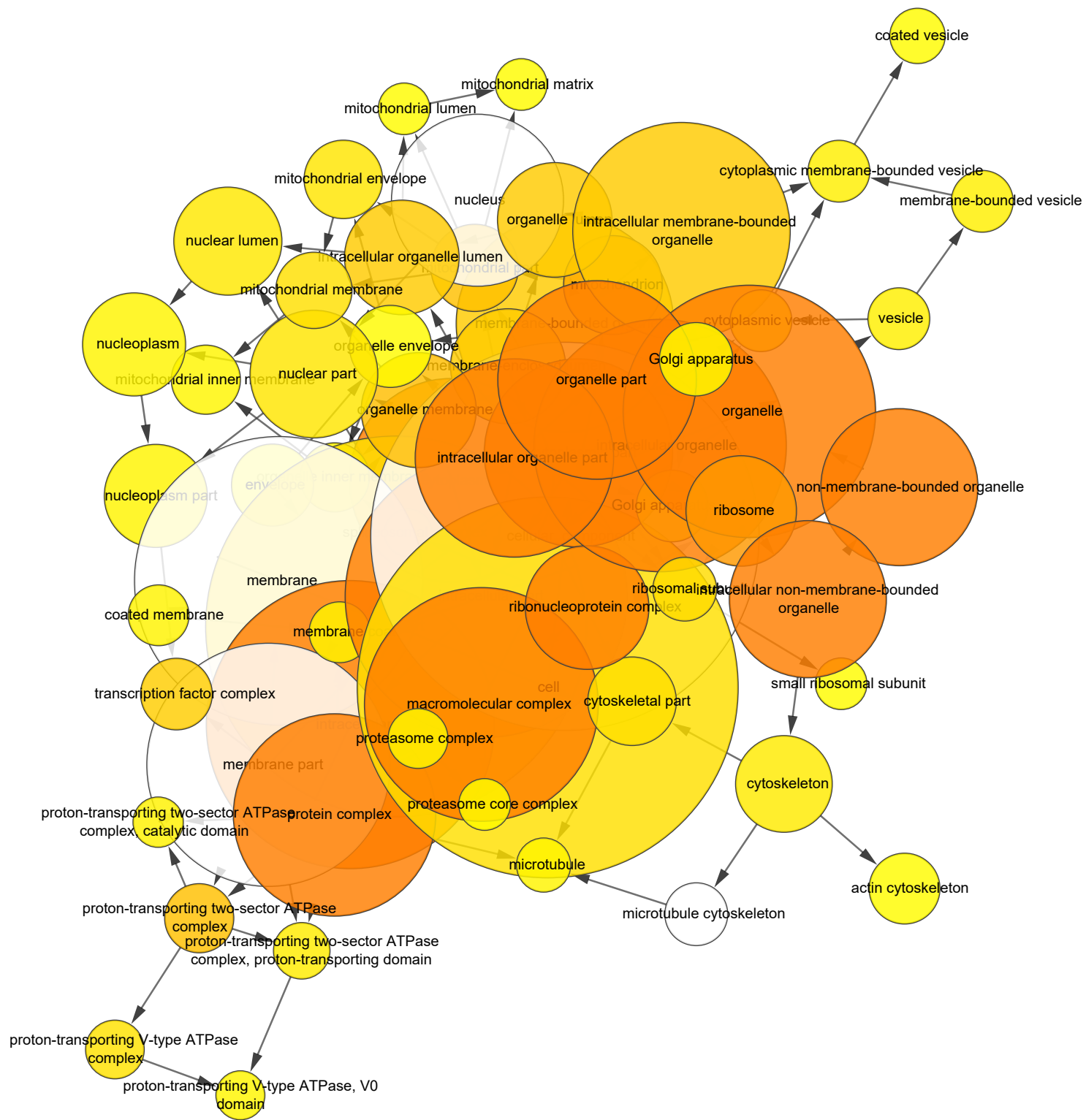

Supplement: Supplementary file 1 [file genes-11-00570-s001.zip › genes-746709-supplementary/Supp-Figure/Figure S4.pdf]

Figure S5

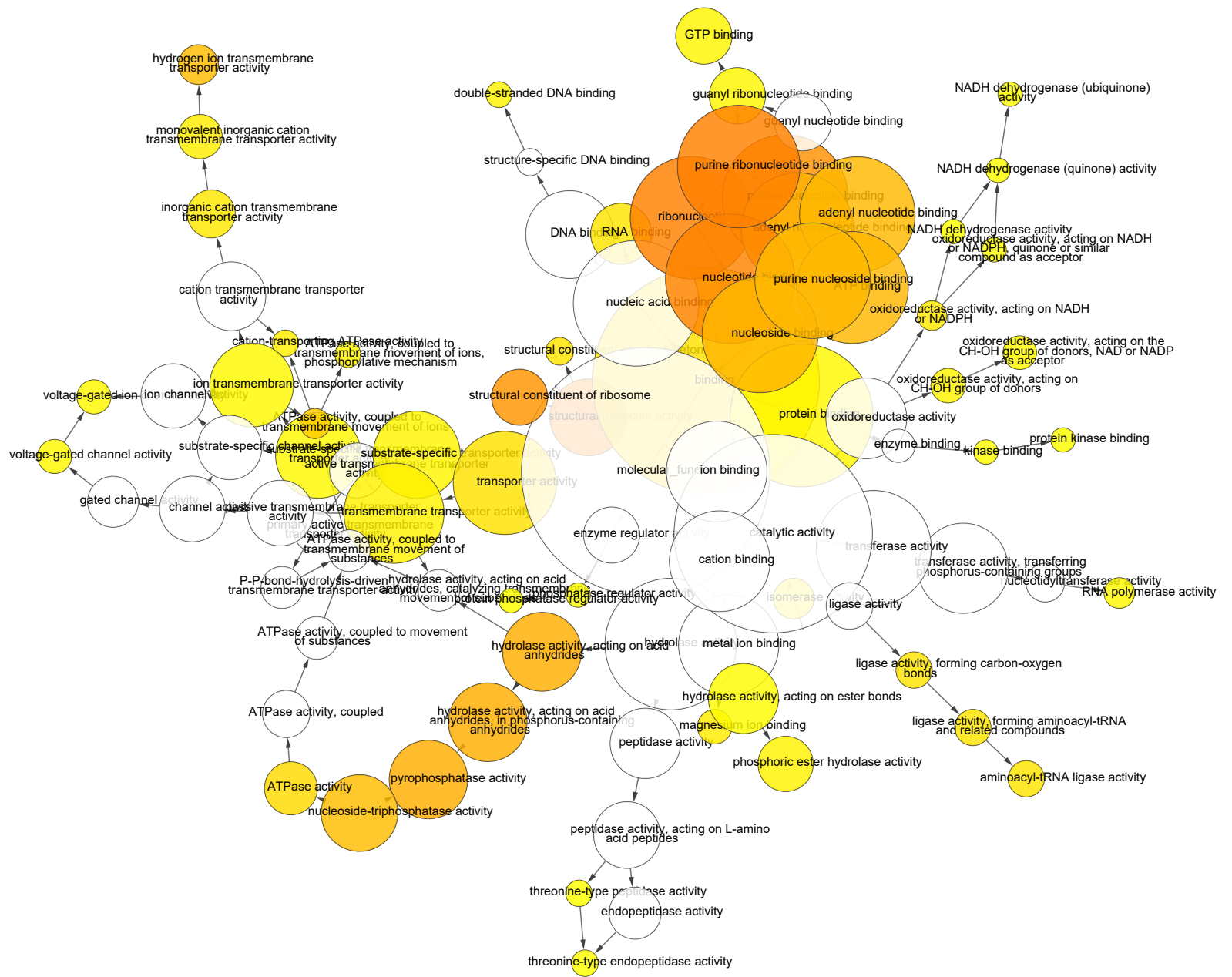

Supplement: Supplementary file 1 [file genes-11-00570-s001.zip › genes-746709-supplementary/Supp-Figure/Figure S5.pdf]
